# Potential of Covalent Organic Framework in Pharmacy and Biomedicine: Influence of Structure on Properties and Applications

**DOI:** 10.1002/cmdc.202501039

**Published:** 2026-05-24

**Authors:** Julia Kolodziejczyk, Marta E. Plonska‐Brzezinska

**Affiliations:** ^1^ Department of Organic Chemistry Faculty of Medicine with the Division of Dentistry and Division of Medical Education in English Medical University of Bialystok Bialystok Poland

**Keywords:** biomaterials, biomedical applications, covalent organic frameworks, pharmaceutical potential, porous materials

## Abstract

Covalent organic frameworks (COFs) are a versatile class of crystalline porous materials with growing potential in pharmaceutical and biomedical applications due to their structural precision, modular chemistry, tunable porosity, and emerging biocompatibility. Unlike prior reviews centered mainly on individual applications, this review adopts a structure‐property‐bioperformance‐translation framework to clarify how COF design governs biological function and clinical promise. We analyze how linkage chemistry, topology, pore environment, surface properties, and particle engineering influence protein corona formation, biodistribution, degradation, therapeutic efficacy, and safety. Particular attention is given to translational challenges still under‐represented in the literature, including synthesis under biomedical constraints, scalability, reproducibility, residual solvent and impurity control, sterilization, green chemistry, and regulatory compatibility. We further discuss the expanding roles of COFs in drug and biomacromolecule delivery, photodynamic, photothermal, and chemodynamic therapy, as well as in emerging immunotherapeutic and combination treatment platforms. Antimicrobial, wound‐healing, diagnostic, and imaging applications are also considered, with emphasis on the relationship between framework design and functional performance. Disease‐oriented case studies supported by in vivo evidence highlight both opportunities and current limitations across cancer, infectious, and cardiovascular models. This review outlines key principles for translating COFs into clinically relevant therapeutics and diagnostics.

## Graphical abstract

From modular COF design to biomedical translation, this Review maps how linkage chemistry, topology, pore environment, and particle engineering govern bioperformance, therapeutic and diagnostic function, safety, and clinical readiness.

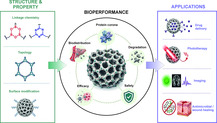

## 1 Introduction

Porous organic networks (PONs) constitute a diverse family of materials composed solely of light elements (C, H, N, O, S, B, and P) that are covalently linked to form frameworks, combining permanent porosity, high stability, and broad chemical tunability [1, 2]. The development of PONs can be traced back to the 1990s, when synthetic strategies were first established to produce permanently porous organic solids without the need for templating or metal coordination [3, 4]. Over time, several major subclasses of PONs have emerged, including hypercrosslinked polymers (HCPs) [5], polymers of intrinsic microporosity (PIMs) [6], conjugated microporous polymers (CMPs) [7], and porous aromatic frameworks [8]. Each subclass is defined by a characteristic structural design principle.

1. HCPs are formed through extensive crosslinking reactions, which generate rigid networks with high surface areas.
2. PIMs exhibit microporosity because their contorted polymer backbones prevent efficient chain packing.
3. CMPs contain π‐conjugated frameworks, making them attractive for electronic and optoelectronic applications.
4. PAFs are constructed from aromatic building blocks and are known for their exceptionally high surface areas and chemical robustness.

These amorphous PONs have established a versatile platform for gas adsorption and separation [9, 10], heterogeneous catalysis [11, 12], energy storage [13, 14, 15], chemical sensing [16], and environmental remediation [17]. However, their lack of long‐range order remains a major limitation, as it hinders precise structural characterization and makes it difficult to establish direct relationships between framework structure and function [18].

A major breakthrough in this field occurred in 2005 with the discovery of COFs [19]. By employing dynamic covalent chemistry, COFs introduced crystallinity into porous organic materials, enabling the creation of predictable architectures, uniform pore environments, and molecular‐level tunability [20, 21]. Unlike amorphous PONs, COFs offer precise structural control that allows for the rational design of frameworks with tailored functions, including controlled molecular diffusion [22], selective host–guest interactions [23, 24], and stimuli‐responsive behavior [25]. These features make COFs especially attractive for biomedical and pharmaceutical applications, where precise control over pore size, topology, and surface chemistry is critical for drug encapsulation [26], targeted delivery [27], biosensing [28], and bioimaging [29]. Since their discovery, COFs have undergone rapid evolution, with new generations incorporating biocompatible linkages [30], enhanced hydrolytic stability [31], and functional motifs designed explicitly for therapeutic and diagnostic applications [32].

The transition from amorphous PONs to crystalline COFs represents a major conceptual shift in the design of porous organic materials. Crystalline COFs offer distinct advantages that make them particularly attractive for biomedical applications. Because they are crystalline yet entirely organic, COFs can combine structural order with favorable biocompatibility, low intrinsic toxicity, and good stability under physiological conditions [33, 34]. The absence of metal ions further reduces the risk of metal‐related toxicity or immunogenicity. In addition, their tunable porosity supports high drug‐loading capacity and controlled release behavior, which can improve therapeutic efficacy while reducing side effects [35, 36].

It is also important to distinguish COFs from other porous materials, especially metal–organic frameworks (MOFs), which are also widely investigated for biomedical applications [37, 38]. While MOFs are built from metal nodes and organic linkers, COFs are composed entirely of light elements connected through covalent bonds, resulting in fully organic, metal‐free frameworks. This fundamental difference strongly affects their biomedical profile. COFs generally offer a lower risk of metal‐associated toxicity, high chemical stability, and broad opportunities for molecular‐level structural tuning, depending on the linkage chemistry. In contrast, MOFs often provide greater coordination diversity and may crystallize more readily, but they can also present challenges related to metal ion release and long‐term biocompatibility. These differences are particularly important in the design of materials for pharmaceutical and biomedical use.

**FIGURE 1:**
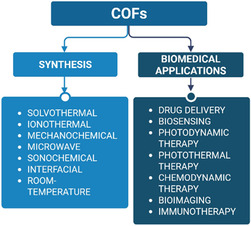
Synthesis methods and biomedical applications of COFs.

In addition, the modular design of COFs allows straightforward post‐synthetic functionalization with biomolecules such as peptides [39], nucleic acids [40], and targeting ligands [41], enhancing biological specificity and therapeutic efficacy. The concept of “structure–property–application” relationships is central to the design of COFs for biomedical use. Establishing clear correlations between structural parameters, such as pore geometry, surface area, and chemical functionality, and functional outcomes enable the rational design of frameworks tailored to specific therapeutic or diagnostic goals. This structure‐guided strategy has facilitated the targeted development of COFs for drug delivery [42], bioimaging [43], biosensing [44], and advanced theranostic platforms (Figure 1) [45]. The emergence of structurally ordered, chemically versatile, and biocompatible COFs provides unprecedented opportunities for molecularly engineered platforms in biomedical and pharmaceutical science, a rapidly expanding frontier in functional materials research.

In recent years, a growing number of reviews have summarized the biomedical applications of COFs. However, most of these studies primarily catalog reported applications, placing less emphasis on how structural design governs biological performance and on advancing these materials toward translational use. The present review addresses this gap by adopting a structure–property–bio‐performance–translation perspective. Specifically, we focus on how key structural parameters, including linkage chemistry, topology, pore environment, surface characteristics, and particle engineering, influence nano–bio‐interactions, biodistribution, degradation, safety, and therapeutic efficacy.

Importantly, this review also highlights manufacturing and pharmaceutical considerations that are often underrepresented in the current literature, including synthesis under biocompatible conditions, scalability, reproducibility, analytical validation, impurity control, residual solvent management, sterilization strategies, and regulatory relevance. By integrating molecular design, biological performance, and translational requirements, this review goes beyond a conventional application‐based summary. Its novelty lies in bridging framework chemistry with pharmaceutical development and process considerations, thereby providing a more comprehensive roadmap for advancing COFs from laboratory materials to clinically relevant nanomedicine platforms. This integrated perspective is particularly timely and significant for the future development of COF‐based systems in pharmaceutical and biomedical science.

## 2 Chemistry of COFs Most Relevant to Biomedical Use

### 2.1 Building Blocks and Linkage Chemistry of COFs

**FIGURE 2:**
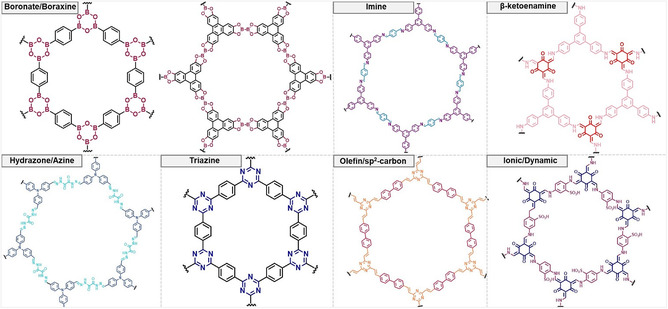
Linkage types of COFs.

The development of COFs critically depends on the choice of linkage chemistry, which governs both the ease of framework assembly and the long‐term stability of the material. Early COFs based on boronate ester linkages demonstrated the feasibility of constructing crystalline porous networks; however, their susceptibility to hydrolysis limited their practical use under physiological or humid conditions [19]. To address this limitation, subsequent generations of COFs employed alternative linkages such as imine [46], β‐ketoenamine [47], hydrazone [48], triazine [49], and sp^2^‐carbon bonds [50], each designed to improve chemical robustness while maintaining sufficient reversibility for crystallization. In parallel, ionic and dynamic covalent linkages were explored to introduce greater structural flexibility and stimuli‐responsiveness (Figure 2) [51].

Table 1 summarizes the most common COF linkages, highlighting their relative stability and degree of reversibility. Early boronate ester and boroxine COFs achieved excellent crystallinity due to their reversible condensation reactions but suffered from poor aqueous stability [52, 54]. Imine‐based frameworks offered improved durability while retaining partial reversibility, though hydrolysis in acidic media remained problematic [55]. The introduction of β‐ketoenamine linkages marked a significant advance: tautomerization stabilized the C=N bond, resulting in frameworks with exceptional resistance to acids, bases, and water [56, 57]. Hydrazone and azine linkages provided intermediate stability, maintaining dynamic reversibility during synthesis while enhancing robustness [58, 59]. In contrast, triazine [60, 61], and sp^2^‐carbon linkages yield highly robust [62, 63], chemically inert frameworks. However, their irreversible formation often limits error correction, reducing crystallinity. Meanwhile, ionic and supramolecular dynamic linkages have been pursued for flexible and stimuli‐responsive architectures, albeit with lower stability under harsh conditions [64, 65]. The evolution of COF linkage chemistry illustrates a fundamental design trade‐off between reversibility, which promotes crystallinity during synthesis, and robustness, which ensures long‐term stability.

**TABLE 1:** Comparison of linkage chemistries in COFs with respect to reversibility and stability.

Linkage type	Typical reaction/formation	Reversibility	Chemical stability	References
Boronate/boroxine	Condensation of boronic acids with diols	High	Low (hydrolyzes in water)	[19, 52, 53]
Imine	Amine, aldehyde	Moderate‐high	Moderate	[46]
β‐Ketoenamine	Tautomeriziation‐stabilized imine	Lower	High	[47]
Hydrazone/Azine	Hydrazine or hydrazide, ketone/aldehyde	Moderate	Higher than imines	[48, 50]
Triazine	Cyclotrimerization of nitriles	Very low	Very high	[49]
Olefin/sp^2^‐carbon	C=C bond formation (e.g., Knoevenagel condensation)	Irreversible	Very high	[50]
Ionic/Dynamic	Electrostatic/H‐bond interactions	Very high	Low‐moderate	[51]

The dimensionality and topology of COFs, 2D or 3D, strongly influence porosity, stability, and functional performance. 2D COFs are the most extensively studied. They consist of covalent sheets stacked into layered structures, forming 1D channels with tunable pore sizes. This architecture facilitates exfoliation into nanosheets and directional transport properties. However, layer stacking introduces complexity: (i) eclipsed (AA) stacking yields high porosity and enhanced charge transport, while (ii) staggered (AB) configurations offer improved mechanical stability at the cost of long‐range order [66, 67]. In contrast, 3D COFs feature covalent connectivity in all three spatial dimensions, producing frameworks with superior mechanical and chemical stability and complex pore architectures [68]. Although their synthesis and crystallization are more challenging, numerous topologies, including carbon nitride and boracite, have been realized, demonstrating the rich structural diversity achievable in 3D COFs [69].

**FIGURE 3:**
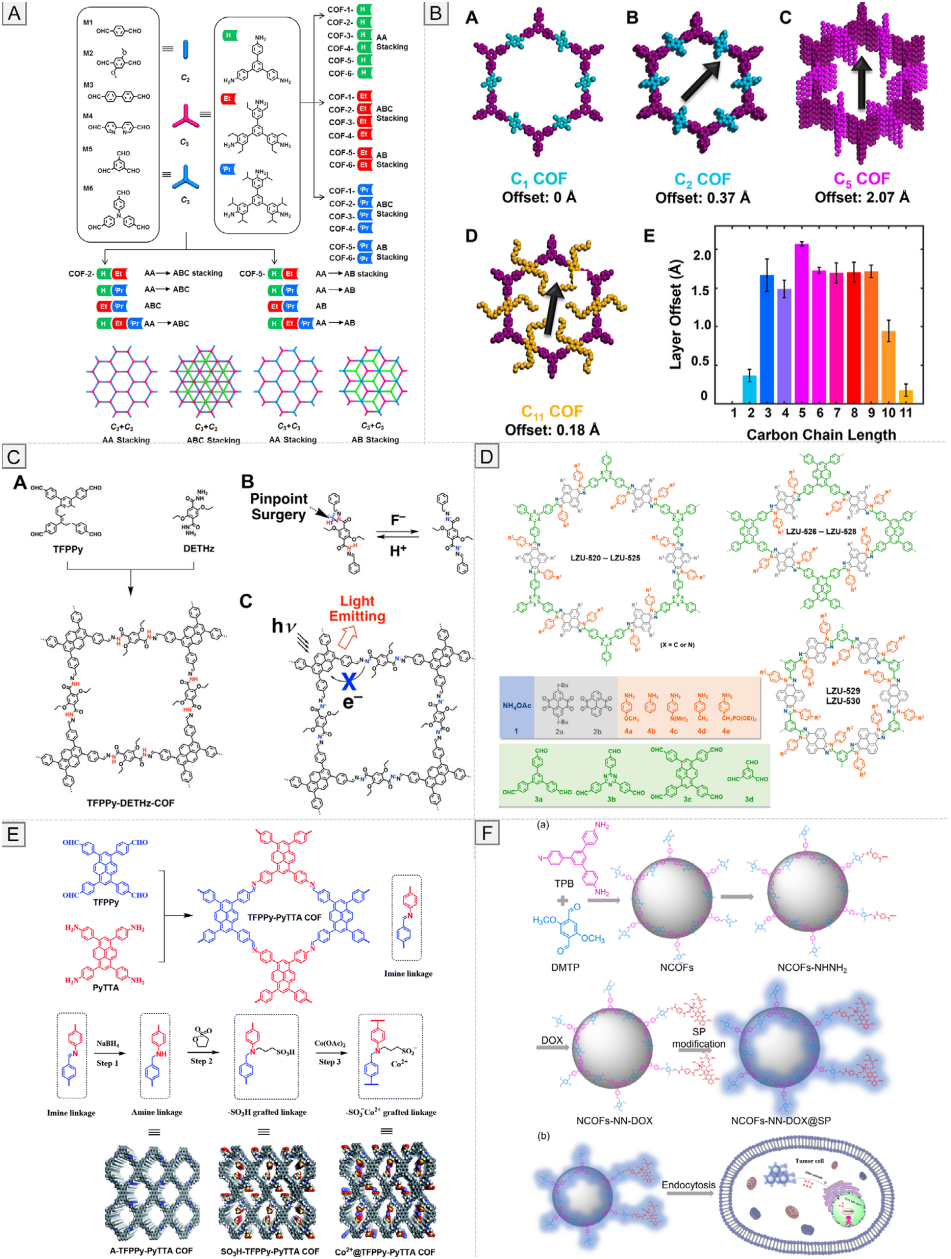
Structural design strategies and functional tuning of COFs. (A) Interlayer engineering. Reproduced with permission from ref. [70]. Copyright 2019 American Chemical Society. (B) Pore design. Reproduced with permission from ref. [71]. Copyright 2023 American Chemical Society. (C) Functionalization of COF skeletons with diverse chemical groups. Reproduced with permission from ref. [72]. Copyright 2018 American Chemical Society. (D) Coordination and adsorption capabilities. Reproduced with permission from ref. [73]. Copyright 2018 American Chemical Society. (E) Pre‐ and post‐synthetic functionalization. Reproduced with permission from ref. [74]. Copyright 2020 Royal Society of Chemistry. (F) Defect engineering and surface modification strategies. Reproduced with permission from ref. [75]. Copyright 2024 Royal Society of Chemistry.

A particularly active research direction involves interlayer engineering in 2D COFs. Functional groups such as –OH, –NH_2_, or –F can mediate hydrogen bonding or electrostatic interactions between adjacent layers, thereby suppressing slippage and enhancing crystallinity (Figure 3A) [70, 76]. Additionally, π–π stacking control and steric tuning have regulated interlayer distances, directly affecting charge transport, optical absorption, and catalytic performance [77]. The dimensionality and stacking topology complement linkage chemistry as key structural design variables. While 2D COFs are promising for applications in electronics, sensing, and membranes, 3D frameworks offer enhanced robustness and complex pore networks [78]. Current efforts focus on engineering stacking interactions to combine the strengths of both dimensionalities, optimizing structure–property relationships for targeted biomedical and technological applications [71].

### 2.2 Pore Architecture and Chemical Microenvironment

The functional performance of COFs is ultimately governed by their pore characteristics, including size, shape, and chemical environment, which dictate molecular access, diffusion, and interaction within the framework. These parameters determine adsorption selectivity, transport efficiency, catalytic activity, and molecular recognition (Figure 3B) [79, 80]. Pore dimensions can be precisely tuned from microporous (<2 nm) to mesoporous (2–50 nm) regimes by varying linker length and node geometry [81]. According to IUPAC terminology, we can also distinguish ultramicropores classified as voids with diameters less than 0.7 nm. In comparison, nanopores are pores that measure between 1 and 50 nm [82]. Longer linkers or larger nodes yield wider pores, suitable for the diffusion of large molecules, whereas compact motifs generate smaller, shape‐selective pores ideal for gas separation or molecular sieving [83]. Pore geometry (cylindrical, hexagonal, triangular, or irregular) also influences accessibility and guest packing [84]. For example, hexagonal channels in 2D COFs promote directional diffusion and uniform molecular flow. At the same time, triangular or irregular cavities create multiple contact points that enhance host–guest interactions, confinement effects, and catalytic or optical properties [85].

Alongside pore geometry, the chemical environment within COF pores plays a crucial role in determining molecular interactions and influencing overall framework functionality. Hydrophobic surfaces promote the adsorption of nonpolar molecules and protect sensitive guests from hydrolytic degradation [86], whereas hydrophilic pores enhance the uptake of polar species [72]. To precisely tune these interactions, a wide range of functional groups, including –COOH, –OH, –SO_3_H, –NH_2_, –NO_2_, –PO_3_, and halogens (–F, –Cl, –Br, –I), have been systematically incorporated into COF skeletons. These functionalized COFs exhibit adjustable electrostatic, coordination, hydrogen‐bonding, and π–π interaction profiles (Figure 3C) [9, 87]. Electrostatic interactions arise from negatively charged groups such as –COOH, –OH, and –SO_3_H, which enable selective adsorption of cationic species, including transition‐metal ions (e.g., Cd^2+^, Pb^2+^) and rare‐earth elements, from aqueous media [88, 89, 90]. Coordination interactions occur through donor sites such as –NH_2_, –COOH, or –PO_3_^2−^, which form stable metal–ligand complexes. For instance, phosphonic acid COFs exhibit strong affinity toward uranium, demonstrating their potential in environmental remediation (Figure 3D) [73, 91, 92]. In addition, π–π stacking interactions between aromatic COF backbones and conjugated guest molecules enhance the adsorption of aromatic pollutants and organic dyes [87]. The hydrophobic–hydrophilic balance, modulated by alkyl or fluorinated substituents, further controls selectivity between polar and nonpolar molecules. This property underpins applications ranging from oil–water separation to controlled drug encapsulation. These tunable chemical features collectively create a versatile molecular sieving environment where electrostatic, coordination, hydrogen‐bonding, and π–π interactions act synergistically to define guest access, orientation, and reactivity. Such molecular precision forms the basis of COF applications in catalysis, sensing, and biomedical systems.

### 2.3 Functionalization, Defect Engineering, and Chemical Modulation

Framework functionality can be further tailored through pre‐ and post‐synthetic functionalization and defect engineering. Pre‐synthetic modification introduces desired functionalities directly into monomers before framework assembly, allowing for precise control over hydrophilicity, charge distribution, and the incorporation of catalytic sites. This strategy enables the creation of COFs with uniform and predictable active sites, which is especially advantageous for biocatalysis and host–guest recognition [93]. Post‐synthetic functionalization, in contrast, modifies COFs after their formation, preserving the underlying topology and porosity. This approach allows incorporation of sensitive functional groups, such as redox‐active centers, fluorescent probes, or biologically relevant ligands, that may not survive the original polymerization conditions [94]. Post‐synthetic methods also enable the tuning of surface chemistry, solubility, and stimuli‐responsiveness, as well as critical parameters for drug delivery, sensing, and catalysis (Figure 3E) [74].

In parallel, defect engineering has emerged as an effective method to fine‐tune COF properties. Defects deliberately introduced or inherent to synthesis can alter porosity, adsorption behavior, and chemical reactivity. Controlled generation of missing linkers or nodes can create additional binding sites, adjust pore sizes, or enhance accessibility to active centers (Figure 3F) [75, 95]. However, excessive or uncontrolled defect formation may impair crystallinity and mechanical strength, emphasizing the importance of maintaining an optimal defect balance to achieve the desired performance. The pre‐ and post‐synthetic functionalization and defect engineering provide a comprehensive design toolbox for tailoring COFs beyond the limitations of their native framework chemistry. Table 2 summarizes representative examples of these approaches, outlining their mechanisms and applications. By integrating these strategies with deliberate control of linkages, dimensionality, and pore characteristics, researchers can produce COFs optimized for selective adsorption, catalysis, biosensing, and targeted drug delivery. These design principles underscore the modularity, tunability, and molecular precision of COFs, positioning them as next‐generation materials for biomedical and pharmaceutical applications.

**TABLE 2:** Summary of functionalization approaches and defect control in COFs.

Strategy	Description and mechanism	Typical functional groups	Advantages	Applications	References
Pre‐synthetic functionalization	Functional groups incorporated into monomers prior to framework formation	‐OH, ‐NH_2_, ‐COOH, ‐F, redox‐active groups	Precise placement, uniform active sites, stable integration	Catalysis, selective adsorption, controlled pore environment, drug loading	[93]
Post‐synthetic functionalization	Chemical modification of the assembled framework	Fluorescent tags, redox‐active moieties, ligands, polymers	Flexible, can introduce sensitive or labile groups, preserves topology	Drug delivery, biosensing, stimuli‐responsive systems, catalytic tuning	[74, 75]
Defect engineering	Controlled introduction or removal of linkers/nodes during or after synthesis	Missing linkers, vacancies, functionalized defects	Creates additional binding sites, adjusts porosity, modulates reactivity	Adsorption enhancement, catalysis, transport modulation, guest access tuning	[95]

### 2.4 Particle Engineering and Morphology, Colloidal Behavior, and Processability

The physical characteristics of COF particles play a decisive role in determining their practical performance. Particle size and morphology influence not only surface area and diffusion kinetics but also framework processability, interaction with guest molecules, and applicability in solution‐based systems [96, 97]. Smaller particles provide a higher surface‐to‐volume ratio, which enhances adsorption kinetics and facilitates more efficient interaction with target molecules or catalysts [98, 99]. Conversely, larger crystals often exhibit superior crystallinity and structural order, which can benefit electronic, optical, or mechanical properties, but may compromise dispersibility and surface accessibility [96, 99]. Morphologies observed in COFs range from spherical, rod‐like, and platelet structures to more complex hierarchical architectures, each affecting packing density, diffusion pathways, and exposure of active sites in different ways.

A particularly transformative strategy in 2D COFs is exfoliation into nanosheets (nCOFs). Exfoliation exposes otherwise buried surfaces, increases accessible surface area, and enhances mass transport, which is critical for catalysis, sensing, and biomedical applications. Nanosheets often exhibit faster reaction kinetics, improved guest molecule adsorption, and enhanced optical or electronic properties compared with their bulk layered counterparts [100]. Techniques for exfoliation include mechanical shear [101], ultrasonication [102, 103], liquid‐assisted delamination [104], and chemical treatments [105, 106], each influencing the thickness, lateral dimensions, and structural integrity of the resulting nanosheets. The degree of exfoliation can be carefully controlled to balance surface exposure with framework stability, allowing for customization in specific applications.

Colloidal stability is another critical parameter, particularly in aqueous environments and biomedical uses. COF particles and nanosheets are prone to aggregation due to van der Waals and π‐π interactions, which can reduce effective surface area, limit diffusion, and hinder uniform dispersion [107, 108]. Stabilization strategies involve the use of dispersants, surfactants [109, 110], or polymer coatings [103, 111] to prevent aggregation, control interparticle interactions [112], and maintain a stable suspension. Functionalization of the particle surface with hydrophilic polymers [113] or charged groups [114] can further enhance stability, allowing COFs to remain dispersed over prolonged periods, which is crucial for applications such as drug delivery, imaging, biosensing, and catalysis.The interplay between particle size, morphology, exfoliation, and colloidal stability is especially relevant for biomedical applications [115]. Exfoliated nCOFs with controlled lateral dimensions can penetrate cells more efficiently. At the same time, surface functionalization and stabilizers can improve biocompatibility [103], control drug loading and release profiles [116], and enhance targeting through electrostatic or hydrogen‐bonding interactions [117, 118]. These parameters govern accessibility of active sites, mass transport, dispersibility, and interaction with the environment, ensuring that COFs and nCOFs perform efficiently in heterogeneous, solution‐based, or biological systems. As such, particle engineering represents a crucial dimension in the rational design of COFs for materials science and biomedical applications.

### 2.5 Stimuli‐Responsive and Hybrid COF Systems

**FIGURE 4:**
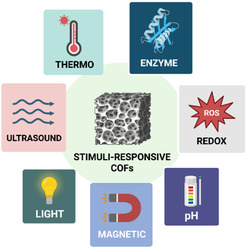
Stimuli‐responsive COFs.

Stimuli‐responsive COFs represent an emerging class of adaptive frameworks that can undergo reversible or controlled changes in response to external triggers. Such materials exhibit dynamic behavior when exposed to pH, redox potential, light, temperature, or mechanical stimuli, allowing precise control over guest uptake, release, and reactivity (Figure 4) [119]. This responsiveness stems from the incorporation of chemical motifs that undergo reversible or irreversible transformations in structure, porosity, or functionality upon exposure to specific environmental signals [120]. These systems offer spatiotemporal control over framework behavior, which is increasingly exploited in drug delivery, biosensing, catalysis, and adaptive materials [121].

**FIGURE 5:**
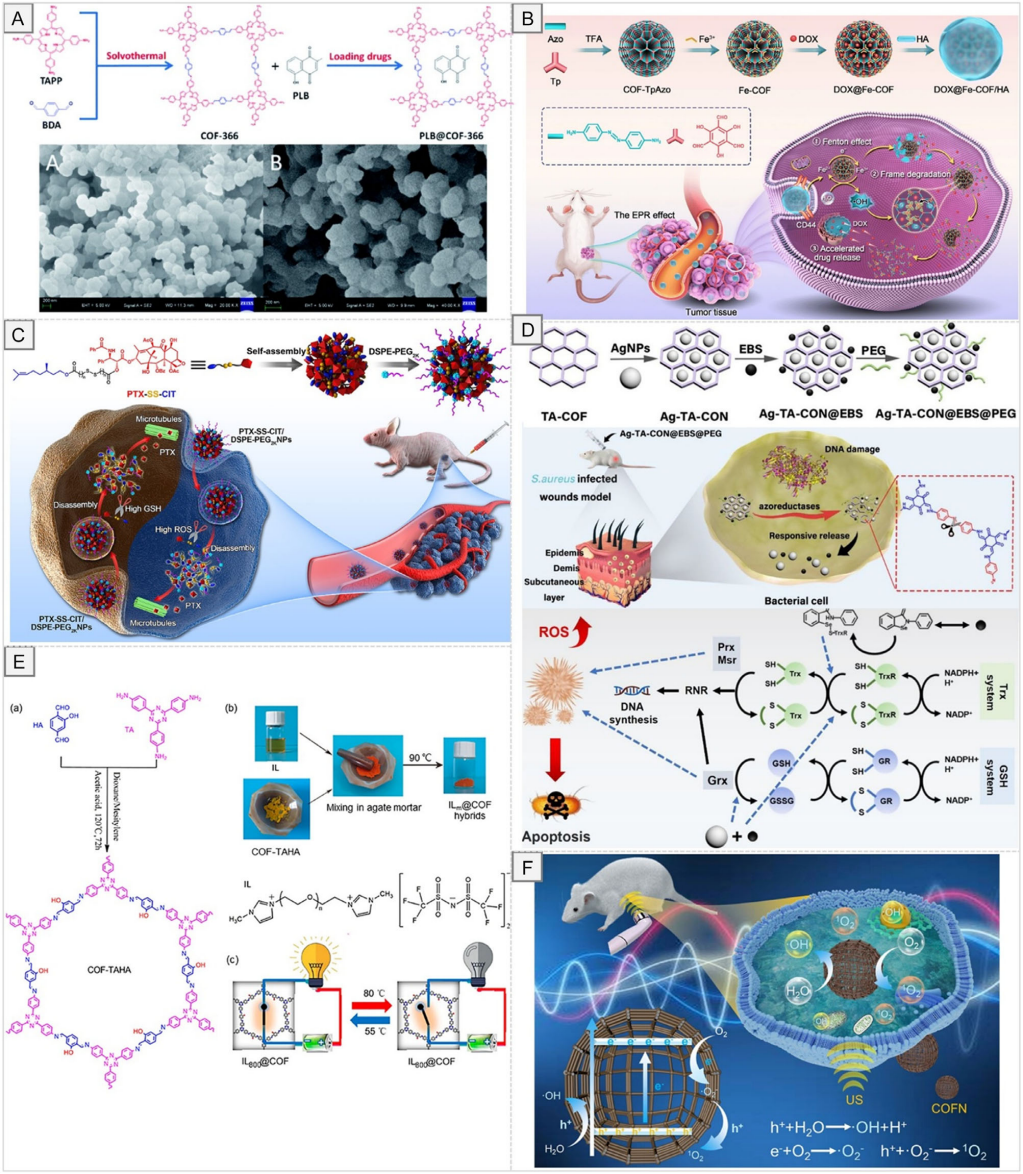
Stimuli‐responsive COFs. (A) pH‐responsive COFs. Reproduced with permission from ref. [120]. Copyright 2022 Royal Society of Chemistry. (B) Redox‐responsive COFs. Reproduced with permission from ref. [119]. Copyright 2023 Royal Society of Chemistry. (C) Redox‐responsive COFs. Reproduced with permission from ref. [122]. Copyright 2018 American Chemical Society. (D) Enzyme‐responsive COFs. Reproduced with permission from ref. [123] Copyright 2022 American Chemical Society. (E) Thermo‐responsive COFs. Reproduced with permission from ref. [124]. Copyright 2023 American Chemical Society. (F) Ultrasound‐responsive COFs. Reproduced with permission from ref. [125]. Copyright 2023 American Chemical Society.

pH‐responsive COFs exploit the protonation or deprotonation of functional groups such as –NH_2_ or –COOH (Figure 5A) [120]. These protonation events modify the local charge, solubility, and pore environment, enabling controlled release of encapsulated drugs under acidic conditions, as found in tumor tissues, lysosomes, or inflamed sites. Such frameworks enable targeted delivery and minimal off‐target effects, exemplified by biodegradable COFs that respond selectively to the tumor microenvironment, facilitating enhanced intracellular drug release and cytotoxicity [121].

Redox‐responsive COFs contain redox‐active linkages or side groups that undergo electron transfer, bond cleavage, or conformational changes under oxidizing or reducing conditions. Typical examples include disulfide bonds and ferrocene moieties, which enable charge release in reductive intracellular environments or reversible switching of catalytic activity (Figure 5B,C) [119, 122, 126, 127]. These frameworks offer precise control over chemical reactivity and molecular transport, which are particularly beneficial for theranostics and catalysis.

Enzyme‐responsive COFs incorporate cleavable linkages or pendant groups that specific enzymes selectively recognize and process. This bio‐orthogonal design allows localized framework activation in response to disease‐associated enzymatic activity, supporting controlled drug release or biosensing of pathological biomarkers (Figure 5D) [123, 128]. Such systems remain inert in healthy tissues but are activated at target sites, offering a promising strategy for smart therapeutic delivery.

Light‐responsive COFs incorporate photoactive chromophores such as azobenzene [129] or porphyrins [130], which undergo reversible electronic or conformational changes upon irradiation. Light exposure modulates pore accessibility, guest binding, or catalytic activity with high spatial and temporal precision. For instance, COF derivatives generate reactive oxygen species (ROS) under near‐infrared (NIR) light, triggering a stepwise degradation of COF and subsequent drug release [131]. Similarly, triazine–porphyrin COFs act as photoabsorbers, producing electron–hole pairs under illumination to drive photocatalytic reactions [132]. These light‐activated systems enable non‐invasive, remote control of framework function, which is relevant for phototherapy, light‐triggered drug release, and optoelectronic devices.

Thermo‐responsive COFs rely on temperature‐sensitive linkages or dynamic bonding motifs that respond to heat by expanding, contracting, or reorganizing the framework. Temperature modulation can influence absorption, charge transport, and flexibility, providing a means to tune material performance. For example, Chen et al. reported a thermally switchable COF where conformational rearrangements control charge transport and optical absorption [133]. Yao et al. demonstrated temperature‐dependent proton conduction in hydrogen‐bonded COFs via reversible restructuring of the hydrogen‐bonding network (Figure 5E) [124]. Similarly, Zhou et al. described COFs with flexible linkers exhibiting reversible lattice breathing and absorption modulation under mild heating [134]. These thermally responsive frameworks provide molecular‐level control over diffusion, sorption, and reaction processes, with implications for sensing, catalysis, and controlled release.

Ultrasound‐responsive COFs utilize acoustic or cavitation effects to induce structural rearrangements or enhance molecular diffusion. This non‐invasive stimulus can trigger the release of drugs, activate catalytic sites, or enhance the dispersion of nanosheets within deep biological tissues. For example, Zhang et al. developed bowl‐shaped COF nanosensitizers loaded with rose bengal and coated with MnO_*x*_, which remain inactive under normal conditions but generate ROS and suppress tumors upon ultrasound exposure [135]. Similarly, Yao et al. reported π‐conjugated COF nanocages that act as highly efficient sonosensitizers, enhancing cavitation and electron transfer for potent in vitro and in vivo sonodynamic therapy (Figure 5F) [125]. Hybrid COF–inorganic composites, such as COF–TiO_2_ systems, amplify sonodynamic activity through ultrasound‐triggered ROS generation and therapeutic release [136].

While pristine COFs exhibit remarkable tunability in linkage chemistry, topology, pore environment, and responsiveness, their integration into composite and hybrid materials offers an additional dimension of functionality. By combining COFs with complementary components such as polymers, biomacromolecules, MOFs, or nanoparticles, researchers have developed materials with enhanced processability, multifunctionality, and application potential in catalysis, energy storage, sensing, and biomedicine. Integrating COFs with polymers is one of the most effective strategies to overcome the mechanical fragility and limited processability of pure COFs. Although COFs are crystalline and highly porous, their brittleness limits their use in flexible membranes or thin films. Researchers have achieved mechanically robust and flexible composites by incorporating COFs into polymer matrices via in situ polymerization, surface grafting, or physical blending. For instance, COF–polymer electrolytes exhibit high lithium‐ion conductivity, where the COF provides ordered ion transport channels and the polymer ensures flexibility and mechanical integrity [137, 138]. Beyond electrochemical applications, COF–polymer membranes have shown excellent potential for gas separation, water purification, and controlled drug release, combining the selectivity and stability of COFs with the processability of polymers [21]. COF–polymer composites are emerging as drug delivery and therapeutic platforms in the biomedical field. Functional polymers, such as polyethylene glycol (PEG), impart biocompatibility and aqueous dispersibility, thereby reducing the aggregation and immunogenicity of COF nanoparticles [103]. These nanocarriers prolong blood circulation and enhance tumor accumulation while enabling controlled drug release. Furthermore, conductive polymers such as polypyrrole can be combined with COFs for dual‐mode chemo–photothermal therapy, where the polymer facilitates photothermal conversion and the COF acts as a high‐capacity drug reservoir [139]. More recently, COF–hydrogel composites have been developed as injectable scaffolds for tissue engineering and localized drug delivery, with the polymer network providing a soft, cell‐compatible matrix and the COF contributing structural stability and drug‐loading capability [140].

Incorporating biomacromolecules, such as proteins, enzymes, DNA, or polysaccharides, into COFs enables the creation of biofunctional hybrid systems that combine biological specificity with structural robustness. The ordered porosity and tunable surface chemistry of COFs create ideal scaffolds for anchoring or encapsulating biomolecules, protecting them from denaturation while maintaining activity. Enzyme–COF composites exhibit enhanced catalytic stability, activity, and recyclability due to confinement effects and controlled microenvironments [141]. DNA‐functionalized COFs stabilize oligonucleotides, enabling selective biosensing and gene delivery, with improved resistance to enzymatic degradation and enhanced cellular uptake [142, 143]. These systems combine the chemical stability of COFs with the biological functionality of macromolecules, creating versatile platforms for targeted therapy, biosensing, and enzyme immobilization.

Combining COFs and MOFs merges the metal‐ligand diversity and tunability of MOFs with the chemical robustness and π‐conjugation of COFs, thereby enhancing their properties. The resulting COF–MOF hybrids display hierarchical porosity, improved stability, and synergistic properties. Early examples demonstrated selective CO_2_ capture, leveraging the robust organic network of COFs and the open metal sites of MOFs [144]. More advanced core–shell architectures (COF@MOF or MOF@COF) enable the spatial separation of functionalities, for example, COF@MOF hybrids where the MOF shell provides catalytic activity. In contrast, the COF core contributes to structural stability and high porosity [145]. Zr–MOF@COF hybrids, for instance, have shown high CO_2_ uptake and excellent catalytic activity in photocatalytic oxidative amine coupling, achieving up to 99% conversion efficiency [146]. COF@MOF hybrids offer exciting prospects for multimodal cancer therapy and theranostic applications in biomedicine.

The MOF component can function as a photosensitizer for photodynamic therapy (PDT), while the COF framework provides drug‐loading capacity, chemical stability, and controlled release. For instance, COF@MOF nanoplatforms have enabled co‐delivery of chemotherapeutic agents and photosensitizers, resulting in synergistic tumor inhibition under light irradiation [147]. Moreover, hybrid systems have been designed for imaging‐guided therapy, where the MOF offers fluorescent or Magnetic Resonance Imaging (MRI) contrast, and the COF ensures prolonged circulation and controlled pharmacokinetics [148]. Integrating COFs into composite and hybrid architectures broadens their scope beyond that of pristine frameworks. Combining COFs with polymers, biomolecules, or MOFs enables precise control over mechanical, chemical, and biological properties, allowing for applications that bridge fundamental materials design with practical biomedical functionality. These hybrid systems exemplify the modularity and versatility of COFs, as well as their capacity to serve as platforms for next‐generation therapeutic, sensing, and diagnostic technologies.

## 3 Structure‐Property Relationships that Govern Bio‐Performance

The integration of COFs with polymers and other hybrid components has enabled the development of advanced biomaterials with improved stability, tunability, and biological performance. However, the successful use of COFs in physiological environments depends fundamentally on understanding the structure–property relationships that govern their behavior in biological systems. In this context, parameters such as pore size, surface area, crystallinity, and chemical functionality are particularly important, because they directly influence biomolecular interactions, drug‐loading capacity, framework stability, and overall therapeutic performance.

Among these factors, chemical functionality plays a central role by determining surface charge, hydrophilicity, and specific intermolecular interactions. These features strongly affect protein adsorption and cellular uptake pathways, which are key determinants of the biological response to nanomaterials [149, 150]. Therefore, elucidating the relationship between structural design and bio‐performance is essential for the rational development of COF‐based biomedical systems.

The stability of COFs in physiological media is one of the most important factors controlling their biomedical applicability. COFs linked by boronate‐ester bonds are particularly susceptible to hydrolytic degradation under aqueous or biological conditions, leading to structural collapse and loss of functionality. Li et al. demonstrated that boronate‐ester hydrolysis in COFs follows unique reaction pathways distinct from those of conventional esters, with higher activation barriers that influence their stability in water [53]. Beyond hydrolysis, oxidative stability is also a critical concern, because ROS present in biological environments may degrade the COF framework. Liang et al. reported that oxidative conditions generated during photocatalytic processes can accelerate COF degradation, underscoring the need to design oxidation‐resistant frameworks for sustained stability and functional performance [151]. These degradation pathways are highly relevant to biomedical applications because they can directly influence drug release profiles, material integrity, and biocompatibility. Structural breakdown may alter release kinetics and generate degradation products that affect biological responses [152]. To overcome these limitations, several stabilization strategies have been investigated. For example, the incorporation of functional polymers has been shown to improve resistance to both hydrolytic and oxidative degradation, thereby enhancing the suitability of COF‐based materials for biomedical use.

### 3.1 Porosity, Crystallinity, and Drug Transport

Porosity, surface area, and pore architecture are central parameters governing the performance of COFs as drug‐delivery platforms. The high surface area of COFs provides a large number of adsorption sites, thereby enhancing drug loading efficiency and facilitating interactions with biomolecules. At the same time, high surface area increases exposure to biological fluids and may therefore promote protein adsorption and protein corona formation, which strongly influence biological identity, cellular uptake, and biodistribution [153, 154]. Thus, surface area is directly linked not only to loading capacity, but also to the in vivo fate of COF particles.

Crystallinity is another important parameter because it determines the degree of structural order, defect density, and framework robustness. In porous materials, higher crystallinity is often associated with lower defect density and more predictable mass‐transport behavior, thereby improving the reproducibility of adsorption and release processes [155]. In the biomedical context, these features are particularly significant because they can affect framework stability in physiological media, degradation behavior, and drug‐release profiles.

The pore size and pore geometry of COFs are especially important for drug transport. These parameters strongly influence molecular accessibility, diffusion pathways, loading efficiency, and release kinetics. Farzan et al. described how mass‐transport mechanisms such as capillary infiltration and diffusion depend strongly on pore structure [156]. Accordingly, pore dimensions determine whether therapeutic molecules can efficiently enter the internal pore system, how they are distributed within the framework, and how rapidly they are released under physiological conditions [157]. If pore size is not properly matched to the cargo's size and physicochemical properties, loading may remain limited to the external surface, resulting in lower encapsulation efficiency and less controlled release. In mesoporous systems, inefficient capillary infiltration can further hinder loading and release, highlighting the importance of designing COFs with pore sizes and pore environments optimized for the intended therapeutic agent.

Drug transport in COFs is also governed by the interplay between pore structure and surface chemistry. Hydrophilic drugs generally interact more favorably with hydrophilic pore surfaces, whereas hydrophobic drugs tend to associate more strongly with hydrophobic frameworks. These interactions affect both loading efficiency and release rate, because they determine the strength of drug–framework binding and the ease of diffusion through the pore network. Therefore, rational tuning of pore size, internal surface polarity, and chemical functionality is essential for achieving predictable drug transport and controlled therapeutic release.

### 3.2 Particle Size and Morphology of COFs: Biomedical Applications

Particle size and morphology are critical design parameters that provide an additional level of control over the biological performance of COFs. Unlike many inorganic nanocarriers, COFs are often obtained as microcrystalline powders with limited control over particle size distribution and shape, which can compromise processability, batch‐to‐batch reproducibility, and biomedical applicability [103, 158, 159, 160]. This limitation is particularly important because particle size strongly influences cellular uptake, tissue access, biodistribution, and clearance pathways in biological systems [161]. For nanoscale delivery systems, particles within an ≈10–200 nm size window are often considered advantageous for systemic administration, although the optimal range depends on material composition and biological context [162, 163, 164]. However, achieving this size regime in COF systems remains challenging, because framework formation is governed by crystallization‐driven nucleation and growth processes that frequently yield polydisperse particles or aggregated microcrystals.

An additional challenge arises from the need to balance nanoscale dimensions with structural order. Synthetic conditions that favor high crystallinity often also promote particle growth, whereas conditions used to suppress crystal growth and reduce particle size may result in lower crystallinity or reduced structural uniformity [164]. As a result, the preparation of COFs with dimensions suitable for biomedical applications requires careful control over nucleation and growth kinetics, which are highly sensitive to solvent composition, monomer concentration, catalyst loading, temperature, reaction time, and interfacial conditions [160, 164].

Particle morphology also strongly affects nano–bio interactions. Spherical particles are often associated with more uniform cellular uptake, whereas anisotropic or high‐aspect‐ratio particles may exhibit different membrane interactions and internalization behavior [163, 165]. In the case of COFs, morphology is highly sensitive to synthetic conditions, and small variations in reaction parameters can lead to substantial changes in particle architecture, including the formation of nanospheres, nanosheets, hollow particles, and other anisotropic structures. Such morphological diversity can be advantageous for tailoring specific functions, but it also introduces challenges in reproducibility, standardization, and scale‐up. For biomedical applications, this is particularly relevant because morphological heterogeneity may translate into variable biological responses, inconsistent uptake behavior, and less predictable in vivo performance.

Several strategies have been explored to improve control over COF particle size and morphology, including room‐temperature size‐controlled growth, interfacial synthesis, emulsion‐based approaches, PEG‐based dispersion strategies, and heterogeneous nucleation routes for hollow structures. These methods have enabled the preparation of nanoscale COFs and morphology‐defined structures with improved dispersibility and biological utility; however, precise, scalable, and reproducible control over COF particle architecture remains limited.

Another major obstacle is the tendency of nanoscale carriers to aggregate in aqueous and physiological media, which can alter effective particle size, reduce colloidal stability, and compromise biological performance [166]. In COF‐based systems, surface functionalization and polymer coatings are therefore often introduced to improve dispersibility and stabilize nanoparticles in biological environments. However, these modifications can also alter surface chemistry and, consequently, biological interactions [166, 167]. Therefore, strategies to control particle size and morphology must also consider their impact on colloidal behavior and surface properties under biologically relevant conditions.

The importance of particle engineering becomes even more evident upon exposure of COFs to biological fluids. Nanoparticles rapidly adsorb proteins and other biomolecules, forming a protein corona that defines their biological identity and strongly influences their in vivo behavior [168, 169]. Because protein corona formation is sensitive to particle size, surface properties, PEGylation state, and particle shape, insufficient control over these parameters may lead to variable cellular uptake, immune recognition, and biodistribution [170]. The surface chemistry of COFs plays a central role in this process. Hydrophilic functional groups, such as –OH and –COOH, preferentially attract proteins such as albumin, whereas hydrophobic groups promote the adsorption of fibrinogen and other plasma proteins. As a result, the chemical functionality of the COF surface largely determines corona composition and, in turn, modulates interactions with cells and the immune system. González‐García et al. demonstrated that surface functionalization directly affects protein corona composition, which subsequently influences immune recognition and the therapeutic performance of nanoparticles [171].

For this reason, strategies to control protein corona formation are highly relevant in the design of COF‐based nanomedicines. Hydrophilic polymers or biomolecular surface layers can reduce opsonization, prolong circulation time, and improve bioavailability. Another promising strategy is the deliberate pre‐coating of COFs with selected proteins to create a more favorable biological identity before systemic exposure. Zhang et al. demonstrated that pre‐coated COFs retained stable stealth properties even after plasma exposure, thereby supporting prolonged circulation and targeted delivery [169]. This example highlights that controlled surface engineering can be used not only to improve colloidal stability, but also to regulate COF–protein interactions in a way that enhances biodistribution, transport behavior, and therapeutic performance. The challenge of controlling particle size and morphology in COFs extends beyond synthetic optimization and directly affects their translational potential in biomedicine. A deeper understanding of how nucleation, growth, aggregation, and surface modification collectively determine biological performance will be essential for the rational design of COF systems with predictable and clinically relevant behavior.

### 3.3 Optoelectronic Properties and Therapeutical Function

COFs have attracted considerable attention for biomedical phototherapy due to their programmable optoelectronic properties. Their modular design enables the incorporation of chromophoric building blocks capable of absorbing light and undergoing photoinduced electronic transitions, leading to the formation of excitons (bound electron–hole pairs). These excitons can migrate through the extended π‐conjugated framework, a process known as exciton migration, which underpins light harvesting, energy transfer, and ROS generation essential for phototherapeutic efficacy. The efficiency of exciton migration in COFs depends on the extent of π‐conjugation, framework rigidity, and crystallographic alignment of building units. Extended π‐conjugation promotes long‐range exciton diffusion, enhancing light‐harvesting and charge–transfer performance. Crystallinity and pore organization also modulate exciton pathways, influencing charge mobility and optical response [172]. Upon light excitation, COFs can generate ROS, including singlet oxygen (^1^O_2_), superoxide (O_2_^−^), and hydroxyl radicals (•OH), which mediate cytotoxicity in PDT. ROS production is governed by the chromophore's electronic structure and the efficiency of charge separation. Frameworks incorporating donor–acceptor motifs show superior ROS yields through facilitated electron transfer.

**FIGURE 6:**
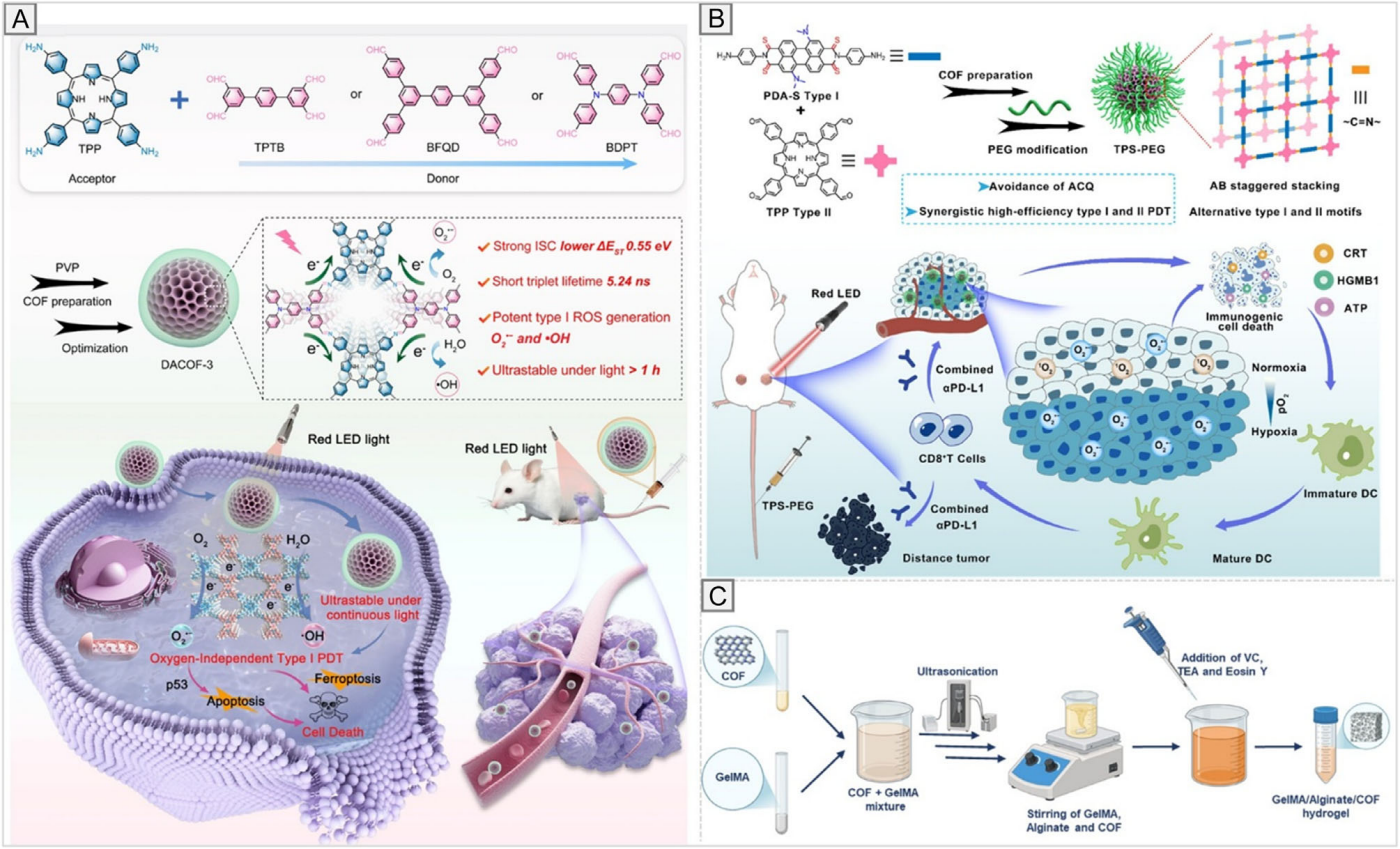
COF‐based systems for phototherapy. (A) Synergistic ROS generation and photothermal heating enable combined PDT/PTT treatment. Reproduced with permission from ref. [173]. Copyright 2025 American Chemical Society. (B) Flexible polyCOFs form robust, elastic membranes. Reproduced with permission from ref. [174]. Copyright 2024 American Chemical Society. (C) COF–hydrogel composites enhance mechanical strength, injectability, and biocompatibility for biomedical applications [175].

Furthermore, porous confinement enhances local oxygen retention and reactant interaction, amplifying ROS generation [173]. Beyond PDT, COFs serve as photothermal therapy (PTT) agents by converting absorbed photons into heat, thereby inducing localized hyperthermia and facilitating cancer cell ablation.Their photothermal conversion efficiency depends on optical absorption intensity and nonradiative energy dissipation. Broad, intense absorption in the NIR range is particularly advantageous for PTT (Figure 6A) [173, 176]. The high surface area and tunable porosity of COFs further boost light absorption and heat generation. Combining PDT and PTT within a single COF framework offers synergistic therapeutic benefits. Such dual‐modality COFs integrate ROS‐induced oxidative damage with photothermal ablation, overcoming tumor heterogeneity and hypoxia‐related resistance (Figure 6B) [174]. The modular nature of COFs allows further functionalization with targeting ligands, metal centers, or imaging agents, creating multifunctional platforms for precision phototherapy.

Translating COFs into biomedical applications requires optimizing their photophysical properties, mechanical resilience, and processability for physiological environments. Conventional COFs are typically brittle and poorly dispersible, limiting their use in injectable or flexible formats. PolyCOFs, which incorporate flexible polymer chains into their backbone, have been developed to address this issue. Wang et al. demonstrated that polyCOFs form freestanding, elastic membranes that retain long‐range order, exhibiting enhanced flexibility and toughness compared with traditional COFs. These materials withstand bending and deformation without fracture, providing a path toward mechanically robust phototherapeutic systems [177].

In parallel, COF–hydrogel composites have emerged as promising injectable and moldable formulations. A recent study reported a gelatin methacryloyl–alginate–COF interpenetrating network, in which the addition of only 1 wt% COF increased the compressive strength sixfold, reduced swelling, and slowed degradation relative to pristine hydrogels (Figure 6C). The composite maintained shape fidelity during extrusion‐based 3D printing and exhibited excellent injectability and flow behavior, enabling minimally invasive administration [175]. From a drug delivery COF perspective, COF–hydrogel systems combine high drug‐loading capacity and controlled release from the porous COF phase with the mechanical adaptability of soft biomaterials. Although quantitative rheological studies on dispersions remain limited, current evidence shows that hybridizing COFs with polymers transforms brittle frameworks into flexible, stable, and biocompatible scaffolds, advancing their readiness for clinical translation.

Beyond photophysical and mechanical optimization, targeted delivery is a critical strategy to enhance COF bio‐performance. Functionalization with biological ligands, such as peptides, aptamers, antibodies, or carbohydrates, enables the specific recognition of diseased cells or tissues, thereby improving selectivity and reducing off‐target effects through multivalent interactions. A representative example is an aptamer‐targeted COF–superparamagnetic Fe_3_O_4_ nanoparticle platform functionalized with a MUC1‐specific aptamer, capable of drug (deferasirox) co‐delivery, MRI imaging, and enhanced antitumor activity in vitro and in vivo compared with nontargeted systems [178]. Similarly, an AS1411‐aptamer‐modified porphyrin COF (pCOF‐I), which is additionally functionalized with PEG and –COOH groups, achieved nucleolin‐targeted chemo‐photodynamic therapy and Computed Tomography (CT) imaging in melanoma models, demonstrating significantly improved uptake and cytotoxicity compared with controls [179]. In receptor‐deficient tumors such as triple‐negative breast cancer, magnetic guidance can complement molecular targeting. A COF@DOX@Fe_3_O_4_ (DOX, Doxorubicin) platform demonstrated improved drug loading, pH‐responsive release, and selective cytotoxicity under magnetic control [180]. In diagnostics, porphyrin‐based COF nanosheet aptasensors functionalized with specific aptamers achieved detection limits in the femtogram‐per‐milliliter range, highlighting the synergistic effect of COF porosity and aptamer–surface interactions in high‐sensitivity biosensing [181, 182]. Multivalent display of targeting ligands on COF surfaces further enhances cell binding and internalization, amplifying therapeutic effects. For instance, aptamer‐decorated COFs exhibited superior binding avidity and cytotoxicity compared to single‐ligand analogs [178, 179]. In another example, a folate‐functionalized COF (FADT‐COF) loaded with Withaferin A displayed selective cytotoxicity toward folate receptor–positive cancer cells, demonstrating ligand–receptor‐mediated targeting [183].

To provide a clearer overview of representative COF‐based systems in biomedical research, selected examples are summarized in Table 3. The table presents their composition, functionalization strategies, and therapeutic applications, thereby illustrating the wide range of design concepts and their influence on biological performance. Together, these examples demonstrate the versatility of COF‐based platforms across diverse biomedical applications, including drug delivery, biosensing, phototherapy, and hybrid biomaterials. They also show that incorporating functional elements, such as targeting ligands, polymers, and photoactive units, enables precise modulation of biological interactions and therapeutic outcomes, underscoring the strong translational potential of COFs for biomedical research.

**TABLE 3:** Representative COFs used in biomedical applications.

COF system	Linkage/composition	Functionalization strategy	Biomedical application	References
PI‐COF‐4/PI‐COF‐5	Imine‐linked COFs	Drug encapsulation in pores	Drug delivery (ibuprofen)	[184]
COF–Fe_3_O_4_ nanoparticle platform	COF and superparamagnetic Fe_3_O_4_	MUC1 aptamer conjugation	Targeted drug delivery, MRI, cancer therapy	[178]
pCOF‐I (porphyrin COF)	Porphyrin‐based COF	AS1411 aptamer + PEG + –COOH	Targeted chemo‐PDT + CT imaging	[179]
COF@DOX@Fe_3_O_4_	COF + Fe_3_O_4_ + DOX	Magnetic targeting + drug loading	pH‐responsive drug delivery	[180]
Porphyrin COF aptasensor	Porphyrin COF nanosheets	Aptamer functionalization	Ultrasensitive biosensing	[181, 182]
FADT‐COF + Withaferin A	Functionalized COF	Folate targeting	Selective cancer therapy	[183]
Phototherapeutic COFs	π‐conjugated COFs	Donor–acceptor design	ROS generation (PDT)	[172, 173]
Photothermal COFs	π‐conjugated COFs	NIR‐absorbing units	Photothermal therapy (PTT)	[176]
Dual PDT/PTT COFs	Hybrid COF systems	Combined photodynamic + photothermal design	Synergistic cancer therapy	[174]
PolyCOFs	COFs with polymer chains	Polymer backbone integration	Flexible phototherapeutic systems	[177]
COF–hydrogel composite	COF in polymer network	Hydrogel incorporation	Injectable drug delivery systems	[175]

Early imine‐linked COFs, such as PI‐COF‐4 and PI‐COF‐5, demonstrated the feasibility of pore‐confined drug loading and controlled ibuprofen release, thereby establishing COFs as promising carrier materials [184]. This concept was later extended by integrating COFs with superparamagnetic Fe_3_O_4_ nanoparticles, enabling magnetically guided drug delivery, multimodal imaging, and cancer therapy [178, 180]. Porphyrin‐based COFs are particularly notable because they combine high cargo‐loading capacity with intrinsic photoactivity. For example, pCOF‐I, functionalized with the AS1411 aptamer, PEG, and carboxyl groups, enabled targeted chemo‐photodynamic therapy together with computed tomography imaging [179]. Porphyrin COF nanosheets have also shown strong potential as ultrasensitive aptasensing platforms [182].

In parallel, functionalized and hybrid COF systems have enabled selective cancer therapy through folate‐mediated targeting [183], as well as advanced phototherapeutic strategies, including photodynamic therapy, photothermal therapy, and synergistic PDT/PTT. These functions are supported by π‐conjugated backbones, donor–acceptor architectures, and near‐infrared‐absorbing building blocks [172, 173]. Polymer‐integrated COFs and COF–hydrogel composites have further broadened the scope of COF‐based materials by providing mechanically adaptable and injectable platforms for phototherapy and drug delivery [175, 177].

These examples highlight the distinctive value of COFs as structurally programmable and multifunctional platforms that can integrate therapeutic delivery, targeting, and diagnostic functions within a single material architecture. Overall, COFs offer a unique combination of structural precision, photophysical versatility, and surface modularity that can be tailored to diverse biomedical needs. Their ability to combine photodynamic and photothermal effects with mechanical adaptability and targeted delivery within one framework underscores their strong potential as next‐generation therapeutic materials. Further progress in understanding structure–property relationships, particularly those linking exciton dynamics, mechanical properties, and ligand‐mediated targeting, will be essential for fully realizing the potential of COFs in precision medicine.

## 4 Synthesis and Manufacturing under Biomedical Constraints

Translating COFs from materials discovery to biomedical application places synthetic strategy and manufacturing control at the core of design. In this context, synthesis governs far more than classical material parameters such as surface area or crystallinity; it also determines particle‐size distribution, defect density, surface chemistry, and residual impurity levels, including trapped solvents, catalysts, and unreacted monomers. These factors, in turn, directly influence batch reproducibility, sterilizability, biocompatibility, and eventual regulatory compatibility. Therefore, when developing COFs for biomedical use, the synthetic route should be assessed not only by traditional materials‐science metrics, such as crystallinity, surface area, and porosity, but also by manufacturing‐relevant criteria, including solvent and energy demand, scalability, process robustness, impurity control, and reproducible colloidal properties.

From a manufacturing perspective, scalability and batch‐to‐batch reproducibility remain major challenges for COFs because framework formation is highly sensitive to reaction parameters such as temperature, solvent composition, monomer concentration, catalyst loading, and reaction time. Even relatively small variations in these parameters can alter crystallinity, particle size, morphology, and defect density, thereby affecting performance and reproducibility in biomedical settings [158, 185]. This sensitivity is evident from mechanochemical and in situ studies showing that catalyst choice, liquid additives, and activation conditions can substantially change reaction kinetics, microstructure, and final porosity [186, 187]. Accordingly, manufacturing strategies for biomedical COFs must aim not only to produce the desired framework, but also to define process windows that yield consistent material attributes across batches.

Scalability is equally important. Classical solvothermal routes often yield highly crystalline materials, but they are often limited by long reaction times, high solvent consumption, and difficulties in standardizing heat and mass transfer at larger scales. In contrast, several newer approaches illustrate possible paths toward more practical production. Ambient aqueous‐phase synthesis has enabled rapid COF formation within 30 min and on a gram scale, with reported yields up to 93% and preparation up to 5.0 g [186]. Likewise, room‐temperature synthesis in CO_2_/water has shown that nanoscale COFs can be obtained under milder and less energy‐intensive conditions [187]. Mechanochemical synthesis further reduces solvent use and is attractive from a green‐manufacturing perspective, while continuous‐flow approaches are particularly promising for scale‐up because they provide tighter control over reaction time, heat transfer, and mixing. Recent continuous‐flow studies demonstrated the production of highly crystalline COFs at rates exceeding 1 g h^−1^ and highlighted flow processing as a route toward more standardized, industrially relevant COF synthesis [188]. Related flow‐reactor work has likewise shown that continuous synthesis can reduce processing time and support integrated production and post‐processing of COFs [189].

Beyond synthesis, manufacturability also depends on the ability to process COFs into robust, application‐relevant physical forms. It remains nontrivial because many COFs are mechanically fragile and can undergo pore collapse or structural damage during pelletization or densification. In this regard, sol–gel processing and monolith formation provide useful examples of how shaping strategies can improve practical handling while preserving accessible porosity. Such approaches are highly relevant for future biomedical manufacturing, where powder handling, formulation, and downstream processing must be tightly controlled [190].

Practically, the synthetic toolbox for COFs encompasses a broad spectrum of methods:

1. High‐temperature solvothermal and ionothermal syntheses yield highly crystalline frameworks but can introduce residual solvents and metal ions.
2. Energy‐assisted techniques, such as microwave or ultrasonic synthesis, reduce reaction times and energy consumption while allowing for tunable particle sizes.
3. Solvent‐free or low‐energy mechanochemical routes minimize environmental impact and facilitate scalable production in the solid state.
4. Interface‐confined and thin‐film growth methods, enabling precise spatial control for coatings or membranes.
5. Emerging aqueous and room‐temperature methods enhance biocompatibility and reduce energy demand and exposure to toxic solvents.

Each synthetic approach leaves a distinctive fingerprint on framework order, defect density, particle morphology, and the type and level of residual contaminants, all of which critically influence biological performance and regulatory acceptance. Importantly, the choice of synthetic method also determines the feasibility of scale‐up and process standardization, with emerging continuous‐flow and mechanochemical strategies offering promising routes toward more reproducible and industrially relevant COF production. In the following section, we summarize and compare these principal synthetic routes, emphasizing their experimental conditions, structural control, and implications for biomedical manufacturing, with reference to representative examples and original literature.

### 4.1 High‐Temperature Solvothermal and Ionothermal Syntheses

Solvothermal synthesis represents the historical foundation and the most widely employed method for constructing COFs. It remains the benchmark against which alternative synthesis routes are evaluated. In its classical form, monomers are dissolved in high‐boiling organic solvents such as mesitylene, *o*‐dichlorobenzene, 1,4‐dioxane, or *N*,*N*‐dimethylformamide and sealed in glass ampoules or Teflon‐lined autoclaves. Condensation reactions are then carried out at elevated temperatures (typically 120–200°C) for 12–72 h. The solvothermal method is highly versatile, offering fine control over morphology, crystallinity, and particle size through variations in solvent polarity, catalyst concentration, and temperature. Acid catalysts such as acetic acid, trifluoroacetic acid, or *p*‐toluenesulfonic acid are commonly employed to accelerate imine condensation, while modulators and co‐solvents can regulate nucleation and growth kinetics. This tunability has made solvothermal synthesis the method of choice for producing highly crystalline COFs, which are suitable for detailed structural characterization by powder X‐ray diffraction (PXRD), solid‐state NMR (ssNMR), and advanced electron microscopy. Indeed, the highest crystallinity and surface areas reported for COFs have been achieved almost exclusively under solvothermal conditions.

**FIGURE 7:**
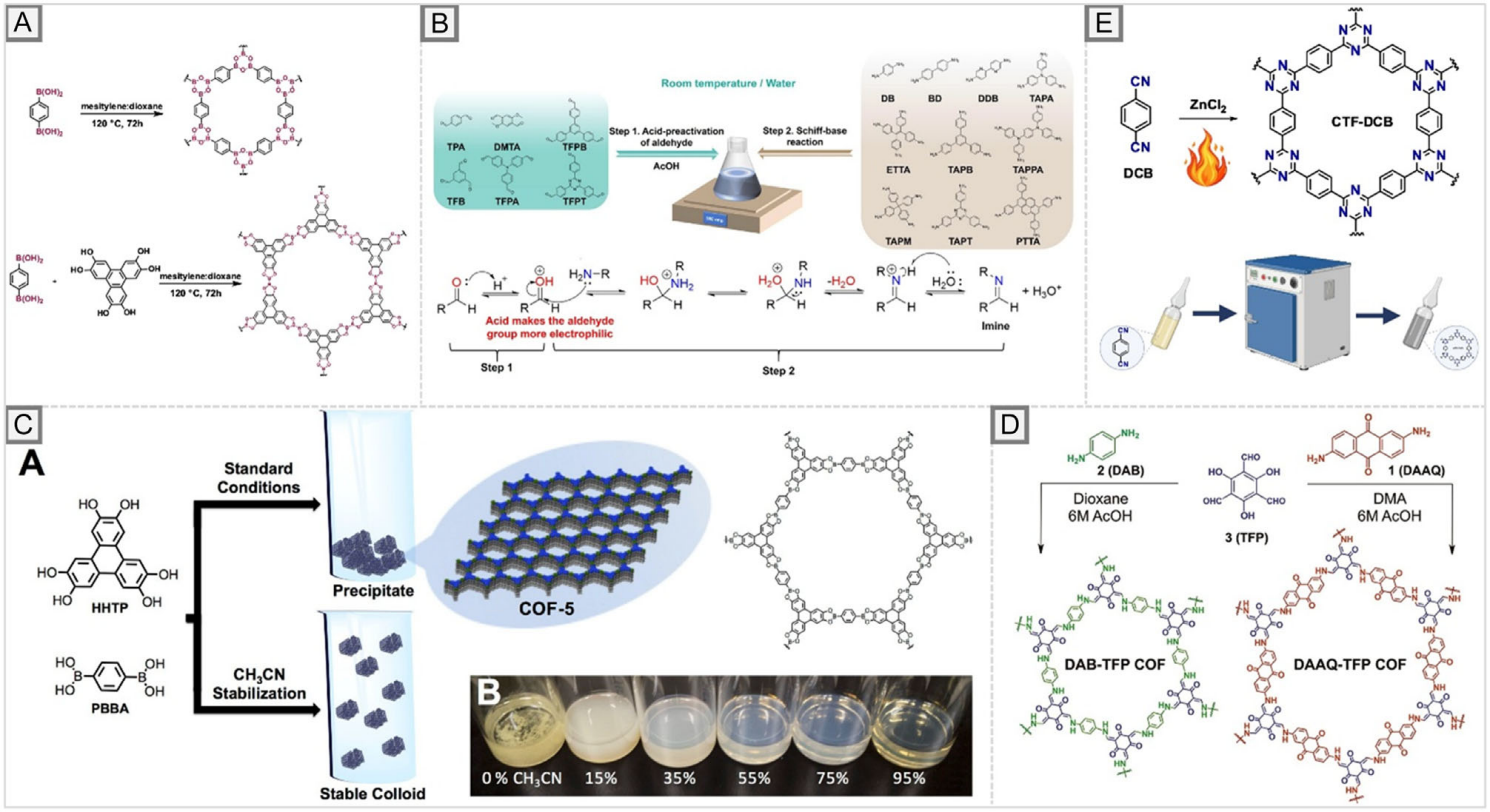
Representative high‐temperature synthesis routes for COFs. (A) Classical solvothermal method [19]. (B) Aqueous solvothermal adaptations. Reproduced with permission from ref. [191]. Copyright 2024 American Chemical Society. (C) Synthesis of boronate ester colloidal COFs. Reproduced with permission from ref. [192]. Copyright 2017 American Chemical Society. (D) β‐ketoenamine COFs with acid stability. Reproduced with permission from ref. [193]. Copyright 2024 American Chemical Society. (E) Ionothermal synthesis of CTFs in molten ZnCl_2_ [49].

The seminal work of Yaghi and co‐workers introduced this approach, reporting the synthesis of COF‐1 and COF‐5 via boronate ester and boroxine formation, thereby establishing the concept of crystalline porous covalent networks (Figure 7A) [19]. The key insight was that reversible condensation, the balance between bond formation and hydrolysis, enabled error correction during framework growth, producing long‐range order in systems otherwise governed by kinetic trapping. This principle was subsequently generalized to other linkages, including imines, hydrazones, and triazines, significantly expanding the structural diversity of COFs [194, 195].

However, the solvothermal approach presents significant challenges from a biomedical manufacturing perspective. The organic solvents typically employed are toxic, poorly biodegradable, and often strongly retained within microporous channels, complicating their complete removal. Even trace solvent residues are unacceptable in pharmaceutical formulations, particularly for injectable or implantable materials, which are subject to stringent regulatory limits on impurities. Furthermore, the high temperatures and long reaction times increase the energy footprint and hinder scale‐up, as autoclave‐based batch processes are inefficient and difficult to standardize. Another concern involves residual catalysts and modulators, which may remain coordinated to the framework and require extensive post‐synthetic purification. Standard protocols include Soxhlet extraction, repeated solvent exchange with lower‐toxicity solvents (e.g., ethanol or acetone), or even supercritical CO_2_ treatment. From a biomedical standpoint, two major issues arise: (i) achieving biocompatibility through the complete removal of toxic residues that could trigger inflammation or cytotoxicity and (ii) ensuring reproducibility in particle size and morphology, which is poorly controlled under solvothermal conditions.

Despite these challenges, solvothermal synthesis remains central to the development of COFs for biomedical applications. For example, imine‐linked COFs prepared via room‐temperature aqueous adaptations of solvothermal protocols have been integrated into cellulose nanofiber@COF nanopapers, exhibiting high mechanical strength, processability, and improved performance in aqueous and biological environments (Figure 7B) [191]. Similarly, boronate ester‐linked 2D COFs have been synthesized as stable colloidal suspensions, overcoming the precipitation issues common to solvothermal products and enabling enhanced dispersibility for potential drug‐delivery formulations (Figure 7C) [192]. Notably, β‐ketoenamine COFs synthesized solvothermally demonstrate exceptional chemical stability, even under strongly acidic conditions, an attribute relevant to tumor microenvironments. DeBlase et al. reported frameworks that maintain their structural integrity and can encapsulate hydrophobic guest molecules under acidic conditions (Figure 7D) [193]. Although initially investigated for energy storage, their resilience under harsh conditions makes them attractive candidates for biomedical carrier design.These examples demonstrate that solvothermal synthesis has limitations, including lengthy reaction times, high energy requirements, and solvent‐related toxicity issues. However, it remains unmatched in producing highly crystalline, well‐defined COFs. Future biomedical progress will rely on adapting solvothermal methodologies through post‐synthetic purification, green‐chemistry optimization, and process control, ensuring materials that combine structural precision with biocompatibility and manufacturing reproducibility.

Among the available synthetic strategies for COFs, ionothermal methods occupy a distinctive position due to their ability to generate highly stable and conjugated architectures under extreme conditions. In this approach, molten inorganic salts act simultaneously as both solvent and catalyst, providing a medium with high polarity, ionic conductivity, and an elevated boiling point. These features enable polycondensation reactions at temperatures far beyond the range of conventional organic solvents, expanding the accessible chemical space toward thermally and chemically resilient frameworks. While early solvothermal COFs established the concept of crystalline porous networks, their stability under acidic or oxidative environments remained limited, an essential consideration for biomedical contexts. Ionothermal synthesis addressed this challenge by enabling the formation of nitrogen‐rich, π‐conjugated frameworks with exceptional robustness and chemical endurance.

A landmark development in this field was the synthesis of CTFs, first reported by Kuhn and coworkers in 2008. These materials were obtained via ionothermal trimerization of nitriles in molten ZnCl_2_ at 400–600°C, yielding microporous, conjugated networks with remarkable stability (Figure 7E) [49]. CTFs resist thermal degradation above 500°C and retain their structure under acidic and oxidative conditions. Within the broader COF family, they represent a subclass defined by triazine linkages and their synthesis under ionothermal conditions, effectively bridging organic framework chemistry with properties characteristic of carbonaceous materials, such as electronic conductivity and catalytic durability.

For biomedical applications, such stability is highly advantageous. When properly engineered, studies have demonstrated that CTFs maintain framework integrity in aqueous and mildly acidic environments. For example, a thiophene‐containing CTF synthesized within mesoporous silica (SBA‐15) exhibited robust aqueous stability, efficient dye degradation, and excellent recyclability during photocatalytic experiments, showing compatibility with conditions relevant to biological media [153, 196]. Further refinement has been achieved through eutectic ionothermal systems, which reduce synthesis temperatures and minimize framework carbonization. For instance, CTF‐ES200, prepared in a NaCl–KCl–ZnCl_2_ eutectic mixture with a melting point near 200°C, exhibited improved optical and electronic properties for photocatalytic hydrogen evolution while mitigating harsh reaction effects [197]. These advances demonstrate that with thoughtful design, ionothermal CTFs can evolve toward biomedically compatible frameworks with controlled composition and functionality. Despite these improvements, several challenges hinder direct biomedical translation. The use of molten salts, particularly ZnCl_2_ in excess, requires exhaustive post‐synthetic purification to remove residual metal and halide ions, as incomplete removal can lead to cytotoxicity or unintended catalytic side reactions. Frameworks produced under high‐temperature ionothermal conditions may also undergo partial carbonization or loss of reactive edge functionalities, thereby limiting post‐synthetic modification and drug‐loading capabilities.

To overcome these limitations, low‐temperature or alternative polycondensation routes have been developed. For instance, CTF‐HUST variants synthesized under milder conditions form layered triazine networks at lower thermal stress, enabling the production of grams of material with reduced impurity levels and demonstrating visible‐light‐driven hydrogen evolution [198]. Likewise, temperature‐dependent studies of CTF‐Py frameworks synthesized at 400°C, 600°C, and 700°C revealed a clear trade‐off: Higher synthesis temperatures enhance porosity and conjugation but increase the risk of carbonization and defect formation, parameters that are critical to biomedical purity, reproducibility, and regulatory compliance [199].

Overall, ionothermal synthesis remains essential for producing robust, conjugated COFs, particularly those requiring high chemical and thermal stability. However, future efforts must focus on reducing residual contamination, minimizing carbonization, and improving framework functionalization under milder ionothermal or hybrid conditions to enable biomedical translation. Such refinements will be pivotal in adapting the unmatched structural stability of ionothermal COFs to the stringent purity and biocompatibility standards of biomedical use.

### 4.2 Solvent‐Free or Low‐Energy Mechanochemical Routes

Mechanochemistry, chemical transformation driven by mechanical force, has emerged as an efficient and sustainable alternative to solvent‐intensive solvothermal methods. In synthesizing COFs, this approach typically involves ball milling or grinding solid monomers in the presence of catalytic amounts of acid, base, or trace liquid additives, a technique known as liquid‐assisted grinding. Mechanochemistry aligns closely with the principles of green chemistry. Compared with solvothermal synthesis, it dramatically reduces solvent use, shortens reaction times from days to minutes, and eliminates the need for high‐temperature, sealed reactors. These attributes are desirable for biomedical applications, where solvent residues represent major regulatory concerns. Minimal solvent use reduces the risk of toxic organic residues trapped in pores, directly improving biocompatibility and purity.

**FIGURE 8:**
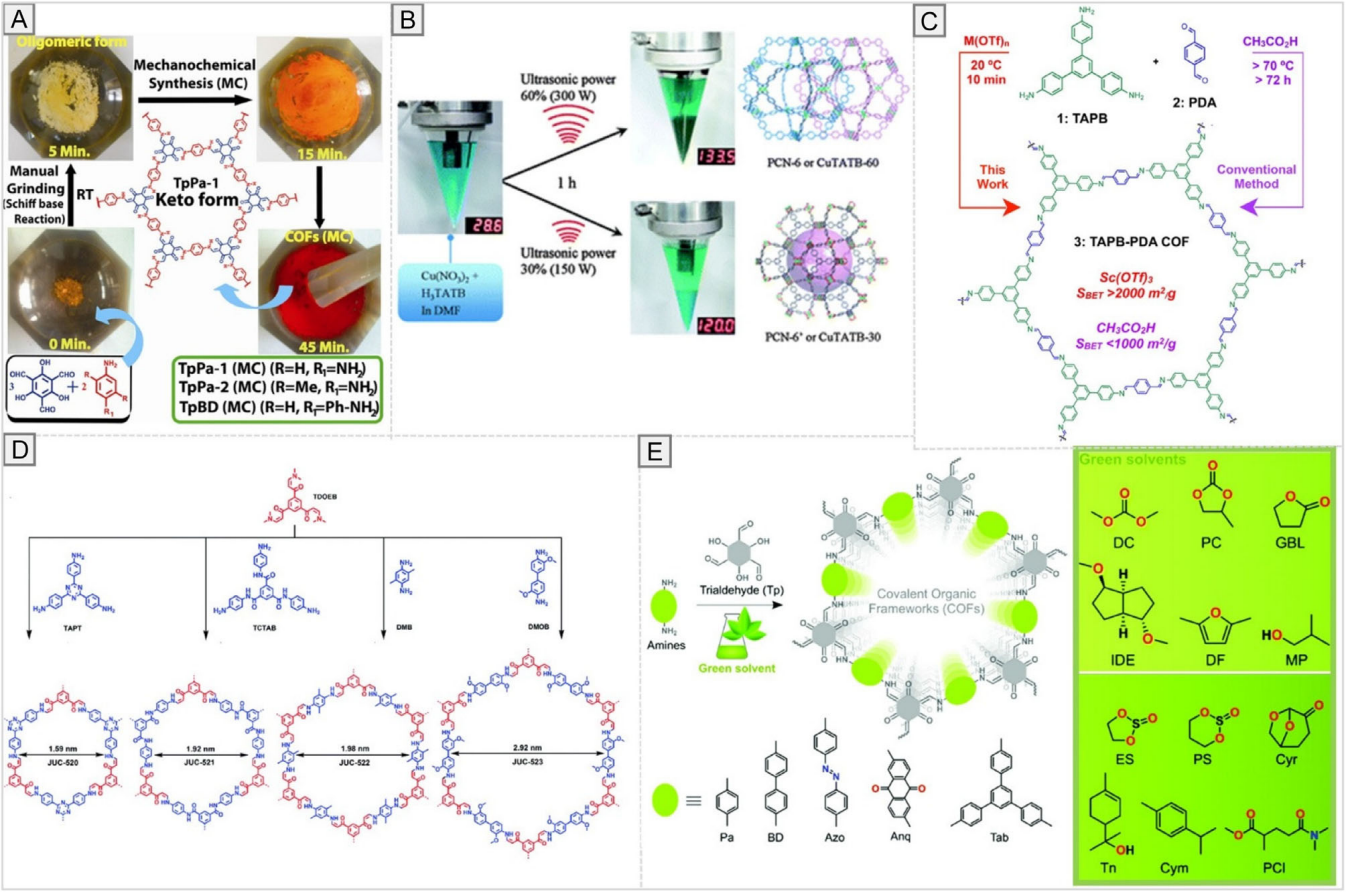
Representative low‐temperature and sustainable synthesis strategies for COFs. (A) Mechanochemical synthesis. Reproduced with permission from ref. [200]. Copyright 2013 American Chemical Society. (B) Sonochemical synthesis. Reproduced with permission from ref. [201]. Copyright 2011 Royal Society of Chemistry. (C) Low temperature synthesis. Reproduced with permission from ref. [202]. Copyright 2017 American Chemical Society. (D) Green/solvent‐free synthesis. Reproduced with permission from ref. [203]. Copyright 2021 Royal Society of Chemistry. (E) Aqueous methods. Reproduced with permission from ref. [186]. Copyright 2019 Royal Society of Chemistry.

The first demonstration of crystalline COFs prepared mechanochemically was reported by Biswal and Banerjee in 2013, who synthesized imine‐linked COFs by grinding 1,3,5‐triformylbenzene with *p*‐phenylenediamine in a planetary ball mill. The reaction produced ordered frameworks without needing high‐boiling solvents or sealed reactors (Figure 8A) [200]. Subsequent studies refined this methodology by optimizing milling frequency, duration, and the nature of liquid additives to enhance reversibility and error correction during bond formation.

These improvements enabled the preparation of boronate‐, hydrazone‐, and β‐ketoenamine‐linked COFs under fully or partially solvent‐free conditions [204]. Mechanochemical synthesis can also yield nanocrystalline or colloidal COFs with tunable particle size, morphology, and aqueous dispersibility, key parameters for drug delivery, imaging, and therapeutic formulations. For example, Biswal et al. demonstrated that solvent‐free grinding produced chemically stable imine COFs under ambient conditions, characterized by significant porosity and resistance to boiling water, as well as strong acid or base, indicating that mechanochemically synthesized COFs can remain stable under harsh environments (Figure 8A) [200]. Building on this, Hamzehpoor et al. recently reported a room‐temperature mechanochemical synthesis of boroxine‐linked COFs, achieving over 20‐fold reduction in solvent use and approximately 100‐fold faster reaction rates relative to solvothermal methods, highlighting the feasibility of this approach even for challenging linkages [205]. Together, these studies establish mechanochemistry as a viable route to formulation‐friendly COFs, offering lower environmental impact and improved compatibility with biomedical manufacturing standards.

Despite these advantages, several challenges remain. Mechanochemically produced COFs often exhibit lower crystallinity than solvothermal counterparts, as the rapid reaction and limited molecular mobility restrict dynamic error correction. Adding small quantities of water or acetic acid can partially restore reversibility; however, achieving long‐range order comparable to solvothermal COFs remains challenging. In many cases, the resulting materials consist of nanocrystals (<100 nm), which poses a limitation for structural characterization by XRD, yet is advantageous for biomedical applications where nanoscale carriers facilitate systemic delivery and cellular uptake. Scalability also poses a challenge. While planetary ball mills are well‐suited to gram‐scale synthesis, translating these methods to kilogram or industrial scales requires continuous mechanochemical reactors, which are only beginning to be developed in industrial process chemistry.

Nevertheless, the biomedical potential of mechanochemically synthesized COFs is significant. Their solvent‐free origin, nanoscale dimensions, and compatibility with sensitive functional groups make them excellent candidates for drug delivery, gene therapy, and bioimaging. Moreover, the alignment of mechanochemical synthesis with low‐energy, environmentally sustainable, and Good Manufacturing Practices (GMP)‐compliant manufacturing positions this method as a strong contender to replace solvothermal approaches in the future, provided that reproducibility, crystallinity, and particle uniformity can be consistently achieved.

### 4.3 Interface‐Confined and Thin‐Film Growth Methods

Interfacial synthesis has emerged as a powerful and versatile strategy for constructing COFs. Unlike conventional solvothermal or sonochemical methods, this approach exploits polymerization at the boundary between two immiscible phases, typically an aqueous and an organic layer, where monomers diffuse across the interface and react. This spatial confinement promotes controlled nucleation and ordered framework growth, yielding well‐defined COF films or layers ideally suited for applications in membranes, sensors, catalysis, and biomedical systems [206]. The interfacial environment inherently aligns monomers during polymerization, enhancing crystallinity and uniformity of the resulting structures. Kim et al. demonstrated this by synthesizing imine‐linked 2D COF films through interfacial polymerization of A_2_B_2_‐type monomers, producing free‐standing films with tunable thicknesses (tens to hundreds of nanometers) and lateral dimensions sufficient for device integration (Figure 8B) [201]. Similarly, Lee et al. reported that CTFs prepared at a liquid–liquid interface exhibited narrow particle size distributions and enhanced porosity compared to bulk‐synthesized analogs, confirming that interfacial polymerization is also applicable to nitrogen‐rich triazine‐based COFs [207].

A significant advantage of interfacial synthesis is its ability to introduce functional groups directly during framework formation, thereby eliminating the need for post‐synthetic modification. For example, Yang et al. synthesized photocatalytic COFs via interfacial polymerization, obtaining materials with improved exciton migration and charge‐carrier mobility due to the highly ordered stacking of 2D layers [208, 209, 210]. The mild reaction conditions, ambient temperature, and limited bulk heating preserve photosensitive and biomolecular functionalities, making this technique particularly suitable for integrating biorelevant or light‐sensitive moieties [206].

Despite these advantages, challenges remain. The uniformity of the liquid interface and monomer diffusion rates critically determines the resulting COF films’ thickness, crystallinity, and homogeneity. Therefore, precise control over solvent composition, monomer concentration, and reaction time is essential. Scalability also presents difficulties: maintaining consistent diffusion and growth can become problematic as the interfacial area increases. However, microfluidic‐assisted interfacial polymerization has recently been developed to achieve more uniform, continuous, and scalable film growth, addressing this key limitation [206].

Interfacial synthesis provides a highly controllable and gentle route for producing COFs and CTFs with precise structural definition and functional versatility. Its compatibility with mild, biocompatible conditions, capacity to form thin films or nanosheets, and potential for in situ functionalization make it an up‐and‐coming technique for biomedical, catalytic, and separation technologies. As such, interfacial polymerization complements traditional solvothermal and sonochemical methods by offering a scalable path to structurally ordered and functionally tunable COFs.

### 4.4 Emerging Aqueous and Room‐Temperature Methods

Over the past decade, there has been a notable shift from traditional high‐temperature solvothermal methods to a diverse range of ambient or room‐temperature synthetic routes for COFs. These newer approaches enable energy‐efficient, environmentally sustainable, and faster framework assembly under mild conditions. Broadly, they encompass aqueous or ambient condensations, catalyst‐assisted low‐temperature assembly, mechanochemical or solvent‐free synthesis, electrochemical and microplasma‐driven polymerization, photochemical initiation, and on‐surface or vapor‐assisted growth, each offering distinct trade‐offs but united by the goal of forming robust covalent networks at low energy cost [211, 212].

Among these, ambient aqueous synthesis represents a particularly significant development for biomedical translation, as it eliminates toxic organic solvents and high‐temperature processing. Kong et al. demonstrated that imine‐linked COFs can be assembled entirely in aqueous media at room temperature by fine‐tuning reaction parameters, such as acid concentration, solvent miscibility, and monomer speciation. This strategy yielded highly crystalline frameworks and even freestanding cellulose nanofiber@COF composite papers, which combine mechanical strength, processability, and compatibility with biological environments [191]. Such water‐based synthesis significantly enhances sustainability and aligns COF production with green chemistry principles relevant to pharmaceutical manufacturing.

Catalyst‐accelerated ambient routes provide a complementary strategy for achieving rapid and ordered framework formation. Pioneering work by Matsumoto, Dichtel, and coworkers showed that Lewis acid catalysts, notably metal triflates (e.g., Sc(OTf)_3_), can significantly accelerate imine condensation at low temperature. These catalysts shift the kinetic–thermodynamic balance, promoting reversible bond exchange and error correction under mild conditions. As a result, crystalline COFs can be obtained within minutes to hours, rather than days, without the need for elevated temperatures. Later studies expanded this concept to include metal nitrates and other low‐temperature promoters, enabling control over crystal habit, defect density, and particle morphology (Figure 8C) [202].

Electrochemical and microplasma‐based syntheses rely on electrically or plasma‐generated reactive species to initiate room‐temperature polymerization, offering high reaction rates and scalable process control. A recent study introduced a microplasma electrochemical protocol capable of producing imine‐linked COFs in minutes, with high crystallinity and a significantly increased space and time yield compared to solvothermal synthesis. Because these techniques decouple reaction chemistry from bulk heating, they are particularly well‐suited for continuous‐flow or industrial reactor integration, paving the way for scalable and sustainable COF manufacturing [213].

### 4.5 Scalability and Environmental Constraints in COF Manufacturing

For COFs to transition from laboratory discovery to biomedical use, scalability and environmental footprint must be treated as fundamental design parameters, not optional considerations (Table 4). In contrast to small‐scale academic syntheses, where a single, high‐crystallinity batch suffices, biomedical translation requires reproducible, kilogram‐scale production using pharmaceutical‐grade solvents and reagents, with energy use and waste generation consistent with sustainability and regulatory standards.

**TABLE 4:** Synthesis routes versus scalability/green metrics/sterilization compatibility.

Synthesis route	Scalability	Green metrics (energy/solvent/waste)	Sterilization compatibility
High‐temperature (solvothermal and ionothermal)	Low to moderate‐ batch processes in sealed autoclaves; limited reproducibility and standardization; difficult solvent removal; high purification demand	Poor‐high solvent and energy consumption; toxic organic solvents (mesitylene, DMF, *o*‐DCB); high E‐factor and PMI; potential metal/acid residues (ZnCl_2_, catalysts)	Low‐ residual solvent and catalyst contamination problematic for parenteral use; γ‐irradiation or e‐beam may degrade imine/boronate linkages; supercritical CO_2_ drying helps purification but not sterilization
Mechanochemical (solvent‐free/low‐energy)	Moderate‐gram‐scale easily achieved; continuous mechanochemical reactors (e.g., twin‐screw extrusion) under development for kg‐scale	Excellent: minimal solvent use (low PMI), low waste, low energy demand; aligns with green‐chemistry and GMP principles	Good: minimal residues improve compatibility with γ‐ and e‐beam sterilization; structure stability depends on linkage (imine/β‐ketoenamine more robust)
Interfacial and thin‐film growth	Moderate: scalable via microfluidic or continuous interfacial reactors; suitable for films and membranes	Good: mild, ambient, low‐energy; minimal bulk heating; limited solvent volume; high atom economy	High: mild synthesis preserves biomolecules; thin films tolerate γ‐irradiation and filtration; suitable for coatings and membranes in biomedical devices
Aqueous and room‐temperature routes	High (emerging)‐ scalable, continuous‐flow and electrochemical methods enable industrial adaptation; reproducible under mild conditions	Excellent‐ water or ethanol/water media; low E‐factor, low toxicity; compatible with ICH Q3C solvent guidelines	High‐ minimal organic residues; frameworks generally stable to γ‐irradiation and sterile filtration; compatible with pharmaceutical sterilization standards

Two interdependent factors dominate process design:

1. Choice of solvents and reagents, which determines the purification burden, residual impurity profile, and whether a COF can be classified as parenteral‐ or implant‐compatible.
2. Process energy and architecture (batch versus continuous; thermal versus alternative activation), which control space‐time yield, cost, and the carbon and energy footprint.

Solvent selection is the single most consequential decision for developing green and pharmaceutical‐compliant COFs. Classical solvothermal protocols rely on high‐boiling, often chlorinated or aromatic solvents such as mesitylene, *o*‐dichlorobenzene, 1,4‐dioxane, or DMF. These media facilitate monomer solubility and control nucleation. Still, they are toxic, poorly biodegradable, and strongly adsorbed within COF pores, making their complete removal challenging and an unacceptable risk for implantable or injectable formulations. Recent systematic studies have identified greener solvent alternatives and developed decision matrices correlating solvent polarity, surface tension, and boiling point with COF crystallinity and porosity. Many frameworks can now be synthesized using benign solvents or mixed‐solvent systems that drastically reduce hazardous content while maintaining structural order (Figure 8D) [203].

A particularly transformative trend is the emergence of aqueous or water‐dominant syntheses. Several groups have shown that β‐ketoenamine and even imine linkages can be crystallized in water or ethanol/water mixtures at ambient or mild temperatures by carefully controlling pH, modulators, and surfactants. These aqueous processes enable gram‐scale production, minimize the use of toxic solvents, simplify purification, and, critically, allow for the direct integration of biomolecules or the application of benign post‐synthetic loading (Figure 8E) [191, 186]. The main challenges remain in achieving kinetic control to prevent amorphous precipitation and extending these strategies to less reversible linkages. Nevertheless, their rapid progress signals a viable route to sustainable and pharmaceutically compliant manufacturing.

From a green‐metrics and regulatory perspective, solvent and energy use must be quantified using established process indicators: (i) E‐factor (kg waste per kg product), (ii) PMI (process mass intensity), (iii) cumulative energy demand (kWh per kg product), and (iv) solvent hazard scoring (per GSK or Pfizer solvent guides). Integrating these key performance indicators (KPIs) early in process development enables the prioritization of synthetic routes that meet environmental and clinical criteria. For example, comparing solvothermal, microwave‐assisted, and mechanochemical routes should involve reporting the solvent mass per gram of COF, residual solvent concentration (via headspace GC–MS), energy input per batch, and time‐to‐release for in‐process controls. Such data are essential for life‐cycle and cost analyses, which funding agencies and regulatory authorities increasingly require. Process intensification strategies, including solvent‐recycling loops, continuous‐flow microwave reactors, and twin‐screw extrusion coupled with in‐line quenching or washing modules, offer realistic engineering solutions to reduce PMI and E‐factor at scale. Recent reviews and case studies document the application of these sustainability metrics to COFs and related porous materials, providing a practical roadmap for GMP‐aligned translation [214, 215].

The biomedical translation of COFs relies on chemical innovation, robust manufacturing infrastructure, rigorous analytical validation, and strict regulatory compliance. Production processes must incorporate solvent recovery systems and high‐sensitivity analytical platforms capable of detecting trace levels of residual solvents, metals, and endotoxins. Early integration of these constraints into synthesis design substantially reduces regulatory risk. For instance, limiting solvent choices to those permitted under the ICH Q3C guideline on residual solvents (EMA, ICH Q3C) ensures compliance from the outset. Selecting lower‐toxicity solvents, such as ethanol or isopropanol, both of which are classified as acceptable for pharmaceutical use, simplifies impurity monitoring and process validation.

For particulate COF synthesis, twin‐screw extrusion offers a scalable, solvent‐minimized, and continuous mechanochemical route. Twin‐screw extrusion has been successfully applied to condensation and imine formation reactions, offering an efficient transition from batch to flow mechanochemistry [216, 217]. Where solvothermal routes remain necessary to achieve high crystallinity, supercritical CO_2_ drying and validated solvent‐exchange protocols are critical for removing residual organics. Supercritical CO_2_ processing, already established in the pharmaceutical industry, provides effective solvent removal and particle drying with minimal thermal degradation [218]. Quantification of solvent residues by headspace GC–MS and metal impurities by ICP–MS should be implemented during early development to ensure compliance with pharmaceutical impurity thresholds.

For COFs to advance from proof‐of‐concept to clinical materials, reproducibility must match chemical sophistication. Minor variations in crystallinity, particle size, or surface chemistry can significantly impact drug loading, release kinetics, immune recognition, and biodistribution. Batch‐to‐batch variability is a significant translational barrier, necessitating the use of standardized quality metrics for regulatory approval. PXRD remains the primary method to confirm long‐range order. However, it cannot capture defect density, stacking faults, or amorphous fractions, which influence porosity and accessibility. ssNMR complements PXRD by verifying bond formation, detecting unreacted monomers, and identifying defective linkages [219]. Brunauer–Emmett–Teller (BET) surface area and pore‐size distribution analyses remain routine, but discrepancies between theoretical and experimental values often signal incomplete condensation or pore collapse [19].

For biomedical applications, particle size and surface charge are equally critical. DLS and ζ‐potential measurements characterize hydrodynamic size and surface electrostatics, which govern colloidal stability and circulation half‐life. Ji et al. demonstrated in situ monitoring of COF‐300 colloid formation using DLS and ζ‐potential, showing that cationic stabilization (ζ ≈ +14.35 mV) arises from protonated amine groups [211]. X‐ray photoelectron spectroscopy (XPS) enables the quantification of heteroatoms, catalysts, and adventitious carbon, which influences protein adsorption and the immune response. For ionothermally synthesized frameworks, XPS and ICP–MS are indispensable for confirming the removal of residual ZnCl_2_ or metal salts, which pose significant toxicological risks.

To achieve GMP‐level consistency, each batch must undergo multimodal characterization, including PXRD and ssNMR (structure verification), BET (porosity), dynamic light scattering (DLS) and ζ‐potential (colloidal behavior), thermogravimetric analysis (TGA) (thermal stability and guest quantification), XPS/ICP–MS (elemental composition), and transmission electron microscopy (TEM)/scanning electron microscopy (SEM) (morphology). Applying such standardized analyses across production batches ensures reproducibility and provides quality assurance for regulatory approval.

COFs intended for biomedical use must tolerate sterilization and formulation processes without compromising structure or function. Regulatory bodies, such as the FDA and EMA, require sterilization using validated methods, including gamma irradiation (γ‐irradiation), electron‐beam (e‐beam) irradiation, or sterile filtration, depending on the dosage form. γ‐Irradiation offers deep penetration and reliability but can generate radicals that cleave covalent bonds, degrade sensitive linkages (e.g., imines, hydrazones), and reduce crystallinity [212]. Comparative studies indicate that e‐beam sterilization may induce fewer chemical changes in organic and protein‐based materials [220].

For nanosheet or colloidal COFs, sterile filtration through 0.22 µm membranes is an attractive, low‐impact method, provided that particle size and dispersion stability are controlled. To prevent aggregation during filtration or storage, lyophilization (freeze‐drying) is often used, though freezing and dehydration stresses can damage porous architectures. Incorporating cryoprotectants and lyoprotectants (e.g., trehalose, sucrose, mannitol) helps preserve morphology and re‐dispersibility, as established in nanoparticle formulations [221]. Incorporating such protective strategies into COF formulations will be essential to ensure post‐sterilization integrity. Post‐sterilization quality checks should include PXRD, BET, ssNMR, TEM/SEM, DLS, ζ‐potential, and ICP–MS to confirm the absence of structural degradation and validate compliance with pharmaceutical quality standards.

Transitioning COF synthesis into GMP manufacturing demands comprehensive control over raw materials, impurities, and process traceability. All monomers, solvents, and additives must be of pharmaceutical grade, with certificates of analysis and validated supplier chains. Endotoxin contamination poses a significant risk, as even trace amounts of lipopolysaccharide (LPS) can elicit severe immune responses. Detection should follow pharmacopeial guidelines using Limulus amebocyte lysate (LAL) or recombinant Factor C assays [222]. Additionally, residual catalysts and monomers must be quantified at parts‐per‐million (ppm) or lower levels using ICP–MS and LC–MS, complying with ICH Q3D (elemental impurities) and Q3C (residual solvents) guidance. Trace metals such as Pd or ZnCl_2_, commonly used in COF and CTF synthesis, require rigorous removal to mitigate cytotoxicity.

Ultimately, aligning COF production with GMP standards requires more than chemical optimization; it necessitates dedicated infrastructure, including cleanroom facilities, closed‐system reactors, validated solvent handling, and in‐line monitoring tools for particle size, porosity, and impurity control. Integrating these practices with sterilization compatibility, protective formulation, and comprehensive analytical verification will be essential for transitioning COFs from laboratory‐scale prototypes to clinically viable materials.

### 4.6 Quality Control and Analytical Characterization Techniques

Quality control is another essential requirement for biomedical translation. In addition to framework crystallinity and porosity, it is necessary to monitor residual reagents, catalyst‐derived contaminants, endotoxins, and batch‐dependent changes in particle size, surface charge, and hydrodynamic behavior, as these may influence cytotoxicity, immunogenicity, and pharmacological performance. This need is especially important for COFs prepared under conditions that use organic solvents, acids, metal salts, or post‐synthetic surface modifiers. Consequently, standardized purification, activation, and analytical validation protocols are required to ensure that the final material is reproducible and pharmaceutically acceptable [152]. For biomedical applications, analytical characterization should therefore be viewed not merely as a structural confirmation step, but as a critical component of quality assurance that links framework chemistry to biological function, safety, and translational readiness.

In this context, advanced analytical characterization techniques are essential to establish the structural integrity, purity, and reproducibility of COFs. PXRDremains the primary method for assessing crystallinity, framework periodicity, and phase purity [223], and it is routinely used to compare experimental patterns with simulated structures in order to confirm successful framework formation [194]. In addition, PXRD is highly valuable for detecting structural changes after activation, post‐synthetic modification, drug loading, or exposure to physiological media, making it particularly relevant for evaluating the stability of COFs intended for biomedical use [161].

Nitrogen adsorption–desorption measurements, commonly analyzed by the BET model, provide key information on accessible surface area and porosity [224], whereas pore‐size analysis and adsorption isotherms help determine whether the internal pore system remains open after synthesis, purification, particle functionalization, or cargo incorporation [194]. Because biomedical performance often depends on drug loading capacity, molecular diffusion, and framework accessibility, gas sorption analysis is an essential complement to diffraction‐based structure determination. At the same time, these measurements are highly sensitive to activation conditions, residual solvent, and framework collapse, and therefore also serve as an indirect indicator of sample quality and batch consistency [194, 225].

Spectroscopic methods provide another important level of structural validation. Fourier‐transform infrared spectroscopy (FTIR) is widely used to confirm bond formation and monitor the disappearance of monomer‐specific functional groups [226, 227], while ssNMR offers more direct insight into linkage chemistry, local bonding environments, and chemical conversion within the framework. In particular, ssNMR is highly valuable for distinguishing closely related linkage motifs, confirming condensation efficiency, and detecting residual precursor‐derived signals that may not be obvious from PXRD alone [225]. For this reason, FTIR and ssNMR are best interpreted as complementary techniques: FTIR provides rapid evidence of functional‐group transformation, whereas ssNMR offers a more detailed probe of local framework chemistry.

Microscopy‐based methods are also indispensable for evaluating the physical form of COFs. SEM and TEM enable visualization of particle morphology, aggregation state, and size distribution [228], which are particularly important for biomedical formulations where particle architecture can influence cellular uptake, biodistribution, and colloidal stability [159]. In nanoscale COF systems, electron microscopy is frequently used to verify the formation of nanospheres, nanosheets, hollow particles, or core–shell structures, and to assess whether synthetic processing or surface modification alters particle shape or structural homogeneity [159, 160].

For dispersions intended for biological studies, DLS provides complementary information on hydrodynamic size in aqueous and physiological media [229], while ζ‐potential measurements help assess surface charge and colloidal stability [159]. These measurements are especially important because the particle size observed by electron microscopy under dry conditions may differ substantially from the hydrodynamic diameter in buffered or protein‐containing media. In this respect, DLS and ζ‐potential analysis are highly relevant for predicting aggregation behavior, protein corona formation, suspension stability, and batch‐to‐batch reproducibility under biologically relevant conditions.

TGA and elemental analysis are critical for evaluating thermal stability and for detecting residual solvents, guest molecules, and unreacted monomers, whereas X‐ray photoelectron spectroscopy (XPS) provides valuable information on surface composition, oxidation state, and chemical environment at the particle interface [230, 231]. This is particularly important after post‐synthetic functionalization, drug loading, polymer coating, or incorporation of inorganic components, where surface‐sensitive characterization is needed to verify the success and uniformity of modification. In biomedical COFs, XPS can also help identify residual catalyst‐derived species or unexpected surface contaminants that may influence interfacial behavior and biological response.

To provide a clearer overview of the analytical methods relevant to COF quality assessment, the principal characterization techniques, the information they provide, and their relevance for biomedical translation are summarized in Table 5. This overview highlights that reliable evaluation of COFs requires a complementary, multiparametric analytical workflow rather than reliance on a single method.These observations show that no single analytical method is sufficient to establish COF quality in a biomedical context. Rather, reliable assessment requires a multiparametric workflow that combines structural, spectroscopic, morphological, colloidal, thermal, and surface‐sensitive techniques. Such an integrated approach is essential not only for confirming framework formation, but also for ensuring purity, reproducibility, and pharmaceutical relevance. This is particularly important when COFs are advanced from proof‐of‐concept materials toward translational biomedical platforms, where subtle differences in crystallinity, porosity, particle size, or residual impurities may lead to significant differences in biological performance.

**TABLE 5:** Analytical characterization techniques used to evaluate the structure, purity, and biomedical relevance of COFs.

Technique	Main information obtained	Relevance for biomedical COFs	Main limitations/remarks
Powder X‐ray diffraction (PXRD)	Crystallinity, phase purity, framework periodicity, structural order	Confirms framework formation and monitors structural integrity after drug loading, post‐synthetic modification, sterilization, or exposure to physiological media	Limited sensitivity to amorphous impurities; may not fully resolve local defects
Nitrogen adsorption–desorption (BET analysis)	Specific surface area, pore volume, porosity	Evaluates pore accessibility, drug‐loading potential, and preservation of internal pore structure after activation or functionalization	Strongly dependent on activation conditions; misleading results can occur if residual solvent remains trapped
Pore‐size distribution analysis	Pore dimensions and pore architecture	Important for predicting molecular diffusion, cargo loading, and release behavior	Indirect method; interpretation depends on the model used
FTIR spectroscopy	Functional groups, bond formation, disappearance of monomer signals	Rapid confirmation of framework condensation and post‐synthetic modification	Less specific for detailed local structure; overlapping bands may complicate interpretation
Solid‐state NMR (ssNMR)	Local chemical environment, linkage identity, conversion efficiency	Useful for confirming linkage chemistry and detecting residual precursor‐derived species	Requires specialized instrumentation; less accessible than FTIR
Scanning electron microscopy (SEM)	Particle morphology, surface texture, approximate size distribution	Helps assess particle shape, aggregation, and batch consistency	Dry‐state method; may not represent behavior in biological media
Transmission electron microscopy (TEM)	Internal structure, nanoscale morphology, hollow/core–shell features, particle dimensions	Useful for nanoscale COFs, nanosheets, and hollow particles used in drug delivery	Sample preparation may alter fragile structures; provides local rather than bulk information
Dynamic light scattering (DLS)	Hydrodynamic diameter and colloidal size distribution in suspension	Important for evaluating particle behavior in aqueous or physiological media and predicting biodistribution	Sensitive to aggregation; assumes roughly spherical particles
Zeta‐potential analysis	Surface charge	Useful for assessing colloidal stability, dispersion behavior, and potential interactions with proteins and cells	Strongly affected by medium composition, ionic strength, and pH
Thermogravimetric analysis (TGA)	Thermal stability, residual solvent content, guest molecules	Helps detect trapped solvents, incomplete activation, and framework stability during processing	Does not directly identify the chemical nature of volatile species
Elemental analysis	Bulk elemental composition	Supports compositional verification and detection of incomplete conversion or contamination	Bulk method only; does not provide spatial or surface information
X‐ray photoelectron spectroscopy (XPS)	Surface elemental composition, oxidation state, chemical environment	Useful for surface functionalization, polymer coating, drug conjugation, and impurity assessment	Surface‐sensitive only; may not reflect bulk composition
UV/Vis spectroscopy	Optical absorption, cargo loading in some systems, photophysical properties	Useful for photoactive COFs and for monitoring drug incorporation or release	Provides limited structural information
Fluorescence spectroscopy	Emission behavior, sensing response, cargo tracking	Relevant for imaging, biosensing, and theranostic COFs	Provides mostly functional rather than structural information
High‐performance liquid chromatography (HPLC)	Residual monomers, impurities, released drug quantification	Important for pharmaceutical quality control, impurity profiling, and release studies	Requires extraction and method development
Inductively coupled plasma (ICP‐OES/ICP‐MS)	Trace metal content	Valuable for detecting catalyst residues, metal contamination, or inorganic additives relevant to toxicity	Requires digestion; not informative for framework structure
Endotoxin testing (for example, LAL assay)	Endotoxin contamination	Critical for biomedical translation and in vivo safety assessment	Not a structural method, but essential for biological quality control

## 5 Pharmacology, Toxicology, and Nano‐Bio‐Interactions

The biomedical translation of COFs and related porous nanomaterials requires a systematic understanding of their interactions with biological systems. Unlike small molecules, which are evaluated using established pharmacokinetic and toxicological frameworks, COFs behave as dynamic nanoscale entities. Their size, surface chemistry, porosity, and colloidal stability collectively determine their biological fate. Upon exposure to biological fluids, these materials rapidly interact with proteins and other biomolecules, forming a dynamic “protein corona” that defines their biological identity and governs cellular recognition, uptake pathways, and immune responses [149, 150]. Comprehensive evaluation must therefore integrate in vitro, in vivo, and long‐term studies, linking physicochemical parameters to biological outcomes.

### 5.1 In Vitro Evaluation and Immune Response

In vitro assays are the first step in assessing COF safety. Standard cytotoxicity tests such as MTT and CCK‐8 have been applied to imine‐ and β‐ketoenamine‐linked COFs, revealing cell line–dependent responses. Moderate concentrations (<100 μg/mL) are generally well tolerated by HeLa and NIH‐3T3 fibroblasts, whereas higher doses induce mitochondrial stress and ROS generation [232]. Broader cytotoxicity panels using epithelial, fibroblast, macrophage, and endothelial cell types help define concentration‐dependent viability profiles. For nanomaterials, hemocompatibility and protein corona formation are equally critical. Adsorption of plasma proteins on nanoparticle surfaces can trigger complement activation, platelet aggregation, or hemolysis. Engineered nanomaterials are known to activate the complement cascade, emphasizing the need to tailor surface chemistry to minimize unintended immune responses [233]. The composition of the protein corona is strongly influenced by surface charge, hydrophilicity, and functional groups, and it can evolve through competitive protein adsorption (the Vroman effect), further modulating biological interactions [153]. Activation of the C3a/C5a axis has been linked to acute inflammation in mesoporous silica nanoparticles [234]. In addition to complement activation, nanoparticle‐induced immune responses may involve macrophage polarization, inflammasome activation, and cytokine secretion profiles that depend sensitively on particle size, surface chemistry, and aggregation state [235]. Cytokine release assays using human peripheral blood mononuclear cells (PBMCs) further reveal whether COFs may provoke pro‐inflammatory cascades. Before progressing to animal studies, these in vitro tools enable efficient screening of multiple COF linkages (imine, β‐ketoenamine, triazine).

### 5.2 In Vivo Biodistribution and Long‐Term Effects

In vivo models provide essential insights into the biodistribution, accumulation, degradation, and clearance of compounds. Data from other porous nanomaterials show that particle size and surface charge are key determinants of organ targeting and clearance. Nanoparticles typically accumulate in the reticuloendothelial system (RES), particularly in the liver and spleen, due to the uptake by macrophages [236]. Protein corona formation in vivo further modulates this process by altering opsonization and recognition by phagocytic cells, thereby influencing circulation time and organ distribution [237]. Renal clearance is generally restricted to particles smaller than ~10 nm (hydrodynamic diameter), whereas larger COFs undergo hepatobiliary elimination via bile excretion.

To further elucidate in vivo behavior, the biodistribution of COFs is governed by a complex interplay of physicochemical parameters, including particle size, surface charge, and surface functionalization. Nanoparticles within the size range of ~10–200 nm are typically sequestered by the reticuloendothelial system (RES), leading to accumulation in the liver and spleen. In contrast, smaller particles may partially evade rapid clearance. Surface charge and hydrophilicity, often modulated by polymer coatings such as PEG, influence circulation time and opsonization [162, 238, 239]. In addition, the formation of a protein corona dynamically alters the biological identity of COFs, further affecting biodistribution and cellular uptake [149]. Degradation pathways of COFs in vivo remain an emerging area of investigation and are strongly dependent on linkage chemistry. Imine‐linked COFs may undergo hydrolytic cleavage under acidic or enzymatic conditions, while β‐ketoenamine frameworks exhibit enhanced resistance due to tautomer stabilization [158, 47]. More robust linkages, such as triazine or sp^2^‐carbon bonds, demonstrate limited degradability, raising concerns regarding long‐term persistence. Oxidative degradation mediated by ROS and, in some cases, enzyme‐assisted processes may also contribute to structural breakdown, particularly in functionalized or hybrid systems [240]. Clearance mechanisms are closely linked to both particle size and degradability. While intact COFs are generally eliminated via hepatobiliary pathways, partial degradation into smaller fragments may enable renal excretion. However, systematic studies tracking degradation products and their elimination routes remain limited, highlighting a critical gap for future investigation and clinical translation.

Crossing the blood–brain barrier (BBB) remains a challenging task. Studies on silica and polymeric nanoparticles indicate that ligand functionalization (e.g., transferrin or peptide tags) can enhance endothelial translocation [241]. The metabolic fate of COFs remains underexplored; however, CTFs demonstrate that strong aromatic linkages resist hydrolysis and oxidation, raising questions about the balance between long‐term persistence and biodegradability [242, 243].

Physiologically based pharmacokinetic (PBPK) modeling, as developed for cerium oxide nanoparticles, could provide predictive power once sufficient COF biodistribution data are available [244]. Preliminary in vivo results suggest that active targeting can alter pharmacokinetics: folate‐modified COFs preferentially accumulate in tumor tissue in xenograft models, thereby enhancing DOX delivery and reducing systemic toxicity [245]. Most biomedical COFs are larger than 10 nm and thus follow hepatic clearance rather than renal excretion; systematic pharmacokinetic studies tracking COF degradation products in blood, urine, and feces are urgently needed for regulatory evaluation.

The long‐term fate of COFs is a central concern given their exceptional chemical stability and aromatic backbone. Lessons from inorganic and polymeric nanomaterials indicate that chronic exposure can induce oxidative stress, DNA damage, or immune modulation, depending on size and surface reactivity. For instance, TiO_2_ nanoparticles cause oxidative DNA lesions and mutagenicity in rodents [246], and nanoparticle‐adapted comet assays now enable sensitive detection of such genotoxicity [247].

Reproductive and developmental safety remain understudied for COFs; however, silica nanoparticles have been shown to cross the human placenta in perfusion models [248], raising concerns about potential fetal exposure. Likewise, microbiome interactions are an emerging frontier; oral exposure to TiO_2_ nanoparticles has been shown to disrupt gut bacterial composition and metabolite balance in mice [249]. Analogous studies on COFs are lacking but critical, given possible impacts on host metabolism and immune function. Future work should therefore combine long‐term animal studies with multi‐omics approaches to evaluate systemic, reproductive, and microbiome‐related consequences of COF persistence. Unlike small molecules, for which dose is defined by mass, nanoparticle effects often correlate more strongly with surface area or particle number than total mass [250]. For porous frameworks such as COFs, accessible BET surface area, hydrodynamic size, and ζ‐potential directly influence protein adsorption, drug release, and immune interactions. Importantly, these parameters also determine the extent of opsonization and subsequent immune clearance, linking physicochemical properties directly to immunological outcomes [251]. Incorporating these parameters into dose normalization is essential for pharmacological interpretation and cross‐study comparability. Recent nanotoxicology studies advocate the use of hybrids, such as surface area–normalized or particle‐number–normalized doses, to more accurately reflect biological activity [252]. Establishing standardized dose descriptors early in COF development will facilitate regulatory alignment and data reproducibility, ensuring that pharmacological and toxicological findings can be meaningfully compared across laboratories and formulations.

## 6 Drug and Biomacromolecule Delivery with COFs

COFs have emerged as auspicious materials for drug and biomacromolecule delivery due to their tunable porosity, structural regularity, and chemical robustness. These features enable COFs to encapsulate and protect a broad range of therapeutic agents, including small molecules, peptides, proteins, nucleic acids, and Clustered Regularly Interspaced Short Palindromic Repeat (CRISPR) cargoes, while allowing for targeted and controlled release. Drug loading in COFs is governed by a combination of geometric confinement within permanent pores and a spectrum of host‐guest interactions including: π–π stacking, hydrogen bonding, van der Waals forces, and electrostatic attractions, while mechanistically it can occur via pore encapsulation, adsorption onto functionalized surfaces, or covalent attachment to reactive framework moieties, with each pathway providing distinct loading efficiencies, release kinetics, and stability profiles [253]. Accordingly, the relationship between cargo properties and framework chemistry is central to the rational design of COF‐based delivery systems.

**FIGURE 9:**
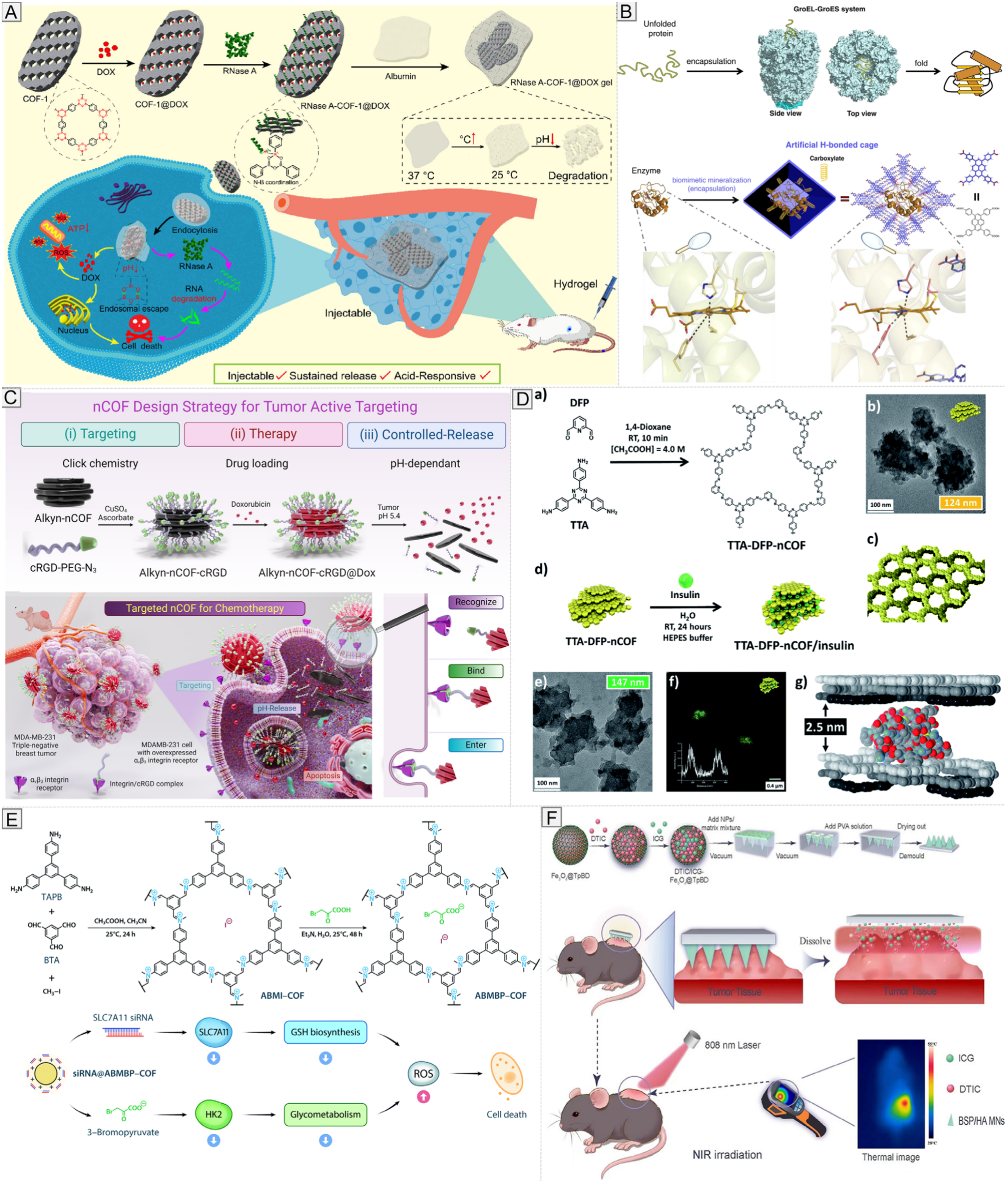
Representative COF‐based delivery systems. (A) Small‐molecule therapeutics and prodrugs. Reproduced with permission from ref. [255]. Copyright 2023 American Chemical Society. (B) Peptide‐ and protein‐loaded COFs for targeted therapy. Reproduced with permission from ref. [256]. Copyright 2022 Springer Nature. (C) Enzyme encapsulation and stabilization. Reproduced with permission from ref. [257]. Copyright 2024 American Chemical Society. (D) Oral insulin formulations. Reproduced with permission from ref. [258]. Copyright 2021 Royal Society of Chemistry. (E) Nucleic acid and CRISPR/Cas delivery. Reproduced with permission from ref. [259]. Copyright 2022 Royal Society of Chemistry. (F) Microneedle platforms for transdermal administration.Reproduced with permission from ref. [260]. Copyright 2023 American Chemical Society.

For small‐molecule therapeutics, COFs offer several advantages over conventional nanocarriers because their well‐defined pore networks permit high loading, reduced premature leakage, and sustained release. In these systems, pore size, pore geometry, and internal surface polarity can be adjusted to match the size and physicochemical properties of the drug. Hydrophobic aromatic drugs are often retained through π–π stacking and hydrophobic interactions with conjugated pore walls, whereas polar or ionizable drugs may be stabilized through hydrogen bonding or electrostatic contacts. This interplay between pore confinement and host–guest chemistry is particularly important because it determines whether the cargo is predominantly localized inside the framework, on the particle surface, or at defect sites, which in turn affects release behavior in physiological media [33]. Representative studies illustrate this principle well. For example, a cage‐based COF was shown to exhibit high drug‐loading capacity and efficient release in simulated body fluid, demonstrating that ordered COF porosity can be directly exploited for small‐molecule transport [254]. For example, Meng et al. demonstrated a COF‐based platform capable of delivering small‐molecule drugs and proteins for lung cancer therapy, establishing proof‐of‐concept for COF nanocarriers in oncology applications (Figure 9A) [255]. In the area of prodrug delivery, COFs improve drug solubility, stability, and bioavailability. Cheetham et al. reported the synthesis of self‐assembling drug amphiphiles, in which hydrophobic anticancer drugs were conjugated to short peptides, resulting in nanostructures with high drug loading and enhanced dispersibility [261]. The modularity of COFs also supports co‐delivery strategies for therapeutics with different physicochemical properties. Because individual pore environments and surface domains can be independently engineered, COFs are particularly attractive for the simultaneous delivery of hydrophobic and hydrophilic drugs, or for combining low‐molecular‐weight drugs with proteins or nucleic acids. In such systems, spatial separation of payloads can reduce premature interactions between cargoes and enable sequential or stimulus‐responsive release. This principle is reflected in existing reports of dual‐compartment or hybrid COF platforms capable of accommodating chemically distinct guests without mutual interference [262]. From a translational perspective, such co‐loading capability is especially valuable because combination therapy frequently requires synchronized yet non‐identical release profiles for maximal efficacy.

COFs are also increasingly recognized as carriers for peptides and proteins, which pose additional delivery challenges due to their large molecular size, conformational fragility, susceptibility to enzymatic degradation, and limited membrane permeability. In this context, biomacromolecule loading can occur through physical entrapment within mesopores, adsorption onto charged or hydrogen‐bonding surfaces, or covalent tethering to functional groups at the framework interface. The nature of the loading mechanism is particularly important for proteins because it influences not only loading efficiency and release rate, but also conformational stability and biological activity. For peptide‐related guest capture, size‐controlled spherical COFs have shown selective enrichment of hydrophobic peptides, highlighting how pore dimensions and hydrophobic interactions can be tuned for biomolecular loading [159]. For proteins and enzymes, Liang et al. showed that in situ trapping of horseradish peroxidase within COF‐LZU1 preserved bioactivity through favorable weak host–guest interactions at the protein–framework interface, thereby illustrating how crystalline porous scaffolds can stabilize fragile biomacromolecules without compromising function [263]. Related advances in hierarchical COF foams further demonstrate that increasing pore hierarchy beyond purely microporous architectures can improve enzyme accommodation, substrate diffusion, and biocatalytic performance, which is highly relevant when larger biomacromolecular cargoes must be loaded efficiently [264].

One of the earliest demonstrations of enzyme encapsulation in COFs was reported by Li et al., who showed that COF channels could preserve enzyme activity and support sustained release, thereby producing stable biocatalysts suitable for both biomedical and industrial applications [265]. Building on this, Chen et al. introduced a related approach using hydrogen‐bonded organic frameworks (HOFs), in which enzyme surface residues trigger framework nucleation to form crystalline scaffolds with ordered mesochannels and remarkable thermal and chemical stability, even under harsh conditions (Figure 9B) [256]. Protein loading can occur through physical entrapment in the pores, adsorption onto charged or hydrogen‐bonding surfaces, or covalent attachment to reactive sites, with each mechanism influencing release rates and protein stability. Although based on HOFs, the work highlights how crystalline porous frameworks can stabilize fragile biomolecules without compromising their activity. Beyond stabilization, COFs have been tailored for targeted protein delivery. Benyettou et al. designed peptide‐modified COF nanocarriers that enhance the targeted delivery and therapeutic performance of small‐molecule drugs in cancer treatment (Figure 9C) [257]. Liang et al. further examined the protein–COF interface, showing that in situ enzyme trapping within COF matrices creates biointerfaces that maintain enzyme conformation and catalytic performance through favorable host–guest interactions [263]. Surface functionalization with polymers or targeting ligands further improves protein loading, protects against aggregation, and promotes uptake by specific cell types, highlighting the versatility of COF design for biomacromolecular delivery. For translational relevance, scalability remains critical. Paul et al. developed a facile, large‐scale synthesis of enzyme@COF biocatalysts thatretained both activity and structural integrity, demonstrating industrially viable routes to COF–protein composites [266]. Moreover, Benyettou et al. engineered a COF‐based oral insulin delivery system, in which the porous scaffold protected insulin from gastric degradation and enabled controlled intestinal release, offering a promising noninvasive alternative to injections (Figure 9D) [258].

The therapeutic efficacy of nucleic acids, including siRNA, miRNA, mRNA, plasmids, and CRISPR/Cas systems, depends critically on efficient cellular internalization and endosomal escape before enzymatic degradation. One of the first demonstrations of COF‐mediated nucleic acid delivery was reported by Zhou et al., who synthesized iminium‐linked, cationic COFs capable of adsorbing and protecting siRNA from enzymatic degradation (Figure 9E) [259]. These frameworks demonstrated efficient cellular uptake and gene silencing in both in vitro and in vivo settings. The authors attributed their efficacy to a proton‐sponge‐like mechanism arising from densely distributed positive charges, which facilitates endosomal escape—a key bottleneck in intracellular delivery. Expanding this concept, Pan et al. designed hollow COF nanostructures to encapsulate CRISPR/Cas12a complexes for intracellular ATP imaging. The COF cavities provided steric protection for fragile CRISPR enzymes and fluorogenic reporters, thereby maintaining their catalytic activity within living cells [267]. Similarly, Lu et al. developed DNA‐functionalized COFs, where covalently attached DNA strands retained sequence‐specific hybridization activity, establishing a foundation for programmable, sequence‐directed delivery and sensin [142]. Recent advances in COF synthesis further broaden their applicability for macromolecular delivery. The seeded growth of single‐crystal 2D COFs, as reported by Ma et al., enables the formation of frameworks with well‐defined internal order and tunable pore dimensions [268]. Complementary progress in combinatorial chemistry has produced hollow and mesoporous COFs suitable for loading larger nucleic acid cargoes such as plasmid DNA and CRISPR ribonucleoproteins (RNPs) [269].

To date, most COF‐based studies have focused on siRNA and plasmid DNA, while robust demonstrations of mRNA or miRNA delivery are still lacking. Moreover, although proton‐sponge buffering and stimuli‐responsive degradation have been proposed to explain endosomal escape, direct mechanistic validation, such as live‐cell imaging of endosomal rupture or biochemical tracking of lysosomal release, is rare. Insights from classical cationic delivery systems remain valuable: the proton‐sponge mechanism described for polyethylenimine by Boussif et al. [270] and more recent mechanistic studies of endosomal escape dynamics provide conceptual frameworks that can inform the future design of cationic COFs [271].

Despite these advances, the loading of biomacromolecules into COFs remains more challenging than the loading of small molecules. Pore aperture must be large enough to permit access, yet sufficient host–guest interactions must still be maintained to avoid rapid leakage. In addition, biomacromolecules often require nanoscale COFs, hierarchical pores, hollow structures, or surface‐mediated immobilization rather than simple micropore filling. This is one reason why particle engineering, pore hierarchy, and surface functionalization are becoming increasingly important in the design of biomedical COFs. These features do not merely improve loading capacity; they determine whether the framework can accommodate the size, flexibility, and interfacial requirements of proteins, nucleic acids, and other complex therapeutic payloads.

The successful clinical translation of COFs in nanomedicine depends on their compatibility with specific administration routes and their ability to overcome biological barriers. Among these, oral delivery remains one of the most challenging yet promising routesof administration. A landmark study by Benyettou et al. demonstrated that imine‐linked nanoscale COFs can encapsulate and protect insulin from gastric degradation while enabling pH‐ and glucose‐responsive release in the intestine. Oral administration of this COF–insulin formulation resulted in a significant reduction in glucose levels in diabetic rats, marking the first successful example of an orally active COF‐based therapy [258]. This study highlights how nanoscale morphology, framework stability, and stimuli‐responsiveness can be tailored to navigate gastrointestinal and epithelial barriers.

Parenteral administration, particularly intravenous injection, remains the most common route in COF‐based cancer therapy. Systemically delivered nanoscale COFs, often PEGylated or biomembrane‐coated, exhibit prolonged circulation, passive tumor accumulation, and potent therapeutic effects in photothermal and chemo‐photothermal modalities [272]. Intratumoral injection has also been widely adopted to achieve high local concentrations, enabling effective tumor ablation upon light activation while minimizing systemic toxicity.

Transdermal delivery is an emerging application area where COFs have been successfully incorporated into dissolving microneedle (MN) platforms. Li et al. designed COF‐based MN patches that provided localized release and combined chemo‐photothermal therapy in melanoma models, achieving marked tumor suppression in vivo (Figure 9F) [260]. These studies demonstrate that COFs can be engineered into minimally invasive delivery devices capable of bypassing the stratum corneum barrier. By contrast, pulmonary or inhalation delivery of COFs remains largely unexplored. While microparticle‐based inhalable powders have shown efficient aerosolization and deep lung deposition, comparable COF formulations have yet to be developed, representing a clear opportunity for future research [273].

The modular chemistry of COFs allows precise integration of stimuli‐responsive linkages and host–guest interactions, making them highly suitable for controlled, site‐specific drug release. Among various triggers, pH‐responsiveness is the most extensively exploited, reflecting the acidic microenvironments of tumors and intracellular compartments. Benyettou et al. engineered a glucose‐ and pH‐responsive COF for oral insulin delivery, achieving selective release under intestinal and diabetic conditions with significant hypoglycemic efficacy in vivo [258]. Similarly, Zhang et al. developed polymer–COF composites enabling dual pH/glucose‐triggered insulin release, confirming the adaptability of COFs for endocrine applications [274]. Redox‐responsive COFs have been realized through incorporation of disulfide (–S–S–) or selenium (Se–Se) linkages, which undergo cleavage in glutathione (GSH)‐rich tumor environments, promoting intracellular drug release. Li et al. reported a disulfide‐linked porphyrin COF that selectively disassembled under high‐GSH conditions, releasing 5‐fluorouracil with high specificity toward cancer cells [119]. Liang et al. demonstrated in situ enzyme encapsulation within COFs, where matrix degradation and release were selectively triggered by biological stimuli, underscoring the utility of enzyme‐sensitive frameworks for therapeutic applications [263].

Light‐triggered release is particularly attractive given the conjugated backbones of many COFs. Wang et al. synthesized porphyrin‐based COFs with strong photodynamic activity under visible light, coupling drug release with ROS generation for synergistic chemo‐photodynamic therapy [275]. Similarly, Li et al. embedded DOX within a COF–MN patch, achieving localized, NIR‐responsive release and effective suppression of melanoma [260]. Emerging work has introduced ultrasound‐responsive COFs. Chen et al. reported that HOFs, a related subclass, can undergo reversible structural changes upon ultrasound exposure, allowing on‐demand drug release via cavitation‐induced mechanical stress [276].

The current studies establish COFs as versatile delivery platforms for both small molecules and biomacromolecules. Their key advantage lies in the ability to combine ordered porosity, modular surface chemistry, controllable host–guest interactions, and stimulus‐responsive behavior within a single material. At the same time, the dominant loading mechanism differs substantially with cargo class: Small molecules are often governed by pore confinement and supramolecular interactions, whereas proteins and nucleic acids more frequently require surface‐mediated binding, electrostatic complexation, hierarchical porosity, or hollow architectures. A clearer mechanistic understanding of these distinct loading pathways will be essential for the rational design of next‐generation COF carriers with predictable release, high retention of biological activity, and improved translational potential.

## 7 Therapeutic Modalities Enabled by COFs

### 7.1 Photodynamic and Photothermal Therapies

PDT relies on light‐activated photosensitizers (PSs) to generate cytotoxic ROS that destroy target cells or pathogens. Upon light irradiation, the photosensitizer is excited from the ground singlet state (S_0_) to an excited singlet state (S_1_), followed by intersystem crossing (ISC) to a long‐lived triplet state (T_1_), which is essential for subsequent ROS generation; therefore, the efficiency of ISC and triplet‐state lifetime are key determinants of PDT performance [277]. COFs provide a modular platform for organizing and stabilizing PS molecules within extended, porous, and often nanoscale architectures, addressing two classical limitations of molecular PSs: aggregation‐caused quenching and poor pharmacokinetics. Embedding chromophores, such as porphyrins, phthalocyanines, BODIPY derivatives, and imidazole‐linked units, into COF backbones or pores enhances ROS production, photostability, and biocompatibility, while enabling combination therapies. For example, porphyrin‐based COF nanoparticles have been formulated to produce singlet oxygen and permit photoacoustic and photothermal imaging, achieving potent in vivo PDT efficacy when appropriately formulated and dosed [275].

ROS formation in PDT typically follows two pathways [278]:

1. Type II, involving energy transfer from the excited triplet PS to ground‐state molecular oxygen, producing singlet oxygen (^1^O_2_). Type II processes dominate under oxygen‐rich conditions.
2. Type I, involving electron or hydrogen transfer, generating superoxide, hydroxyl radicals, or other reactive intermediates. Type I pathways become increasingly relevant in hypoxic environments, enabling oxygen‐independent radical formation.

At the molecular level, these processes are governed by competing energy‐transfer and electron‐transfer pathways, which depend on the redox potential of the photosensitizer and the surrounding microenvironment. In COFs, the extended π‐conjugated framework and ordered arrangement of chromophores facilitate charge separation and reduce nonradiative recombination losses, thereby enhancing ROS generation efficiency compared to molecular systems.

The framework environment of COFs can shift the balance between these mechanisms depending on chromophore identity, oxygen availability, and electronic structure. Luan et al. reported an imidazole‐linked COF optimized for efficient ^1^O_2_ generation, demonstrating that covalent incorporation of tailored chromophores enhances intersystem crossing and Type II efficiency [279]. Similarly, embedding (boron‐dipyrromethene) dyes (BODIPY) or related dyes into nanoscale COFs suppresses aggregation and preserves fluorescence quantum yields, markedly improving singlet‐oxygen generation relative to free dyes [117]. Phthalocyanine‐COF nanosheets follow the same principle: ordered confinement of PS units minimizes self‐quenching and promotes efficient energy transfer to oxygen [280].

A significant limitation of classical PDT is its dependence on oxygen, which reduces efficacy in hypoxic tumors. COF design offers several routes to circumvent this challenge. Dutta et al. developed COF nanocarriers capable of sustaining cytotoxic ROS production under hypoxia by combining singlet oxygen release with oxygen‐independent or catalytic redox pathways [281]. Type I activity can be enhanced by (i) tuning the redox potential of the PS unit, (ii) incorporating redox‐active metals or co‐catalysts into the framework, or (iii) promoting charge separation through extended conjugation or hybridization with inorganic components [282]. Recent work has demonstrated that protonation or metalation of porphyrin‐based COFs can significantly enhance intersystem crossing efficiency, resulting in a higher ROS output under illumination. It illustrates how post‐synthetic modification provides a direct handle to tune PDT activity and the balance between Type I and Type II pathways [283]. A single COF scaffold can therefore be modulated to favor either dominant singlet oxygen production or mixed radical pathways, depending on the desired therapeutic profile [283].

Accurate distinction between Type I and Type II PDT mechanisms in COF systems requires the use of analytical methods. Standard approaches combine chemical probes for singlet oxygen (e.g., DPBF, SOSG) with electron paramagnetic resonance (EPR) spin‐trapping and cellular ROS assays under controlled oxygen conditions. Such protocols, well‐established in photochemistry, have been successfully adapted to COF‐based photosensitizers, enabling a rigorous evaluation of ROS pathways and their biological implications.

**FIGURE 10:**
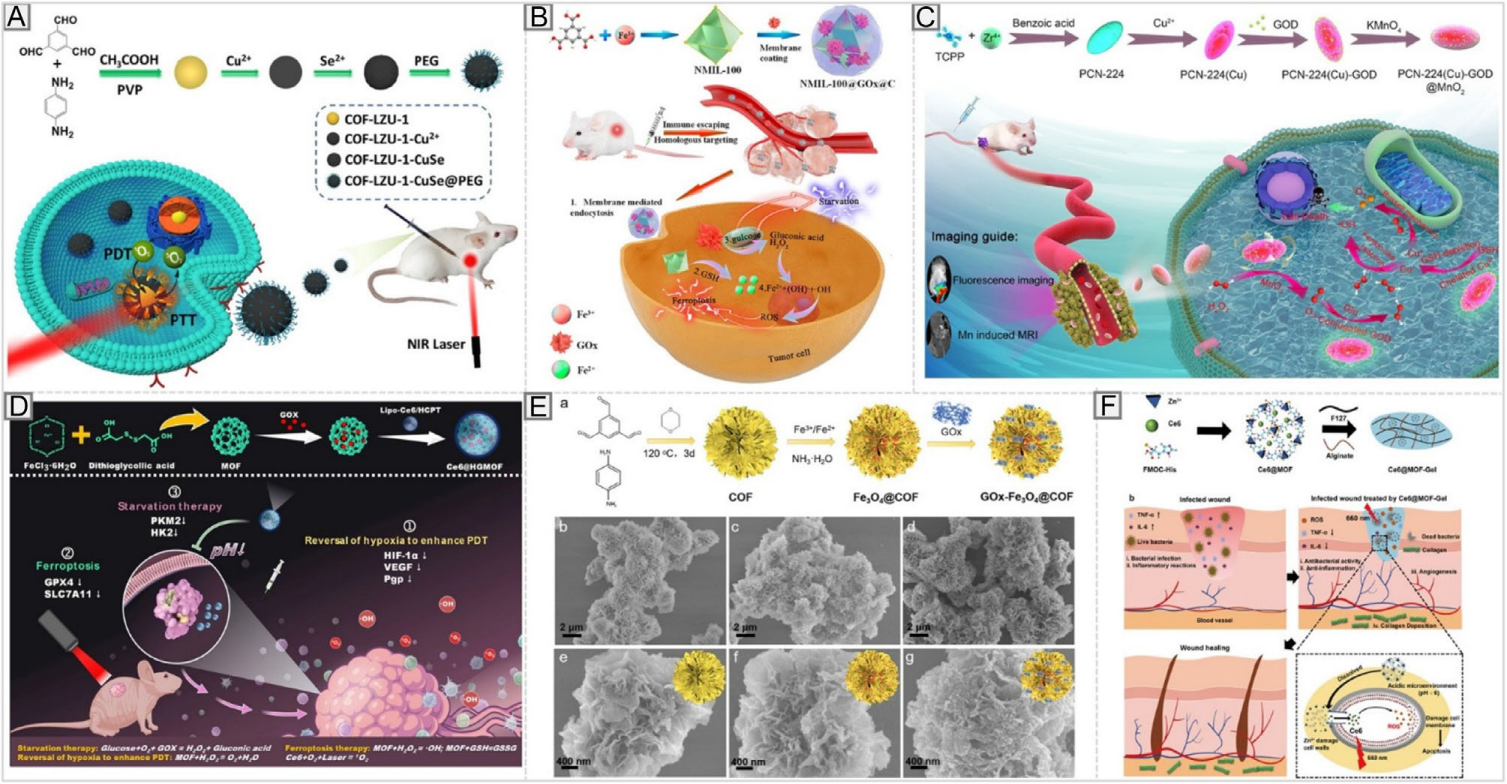
Multifunctional COF‐based therapeutic systems. (A) Photothermal systems. Reproduced with permission from ref. [287]. Copyright 2019 American Chemical Society. (B) Chemodynamic systems. Reproduced with permission from ref. [288]. Copyright 2020 American Chemical Society. (C) Immunoadjuvant systems. Reproduced with permission from ref. [289]. Copyright 2020 American Chemical Society. (D) Catalytic–immune systems. Reproduced with permission from ref. [290]. Copyright 2024 Springer Nature. (E) Antibacterial systems. Reproduced with permission from ref. [291]. Copyright 2023 American Chemical Society. (F) Enzyme‐mediated prodrug activation platforms. Reproduced with permission from ref. [292] Copyright 2023 American Chemical Society.

PTT relies on localized light‐to‐heat conversion to induce hyperthermia and ablate diseased tissues. Mechanistically, PTT conversion arises from nonradiative relaxation of photoexcited states, in which absorbed photon energy is dissipated as vibrational energy (heat) through electron–phonon and phonon–phonon interactions; the efficiency of this process depends on the electronic structure, π‐conjugation, and the availability of nonradiative decay pathways [284]. When combined with PDT, which produces cytotoxic ROS, synergistic phototherapeutic regimens can overcome the intrinsic limitations of each modality, achieving more complete and selective tumor eradication. COFs and COF‐based hybrids have emerged as promising PTT platforms due to their extended π‐conjugation, tunable electronic structure, and modular porosity, which collectively enable strong optical absorption, efficient nonradiative decay (heat generation), and facile incorporation of auxiliary components such as photosensitizers, catalytic centers, or metal and chalcogenide nanoparticles. These features make COFs uniquely suited for multimodal phototherapy and image‐guided cancer treatment. Early studies established that extended π‐conjugation and donor–acceptor (D–A) motifs significantly enhance NIR absorption and photothermal conversion efficiency. Purpose‐built D–A COFs demonstrate how backbone engineering dictates optical and thermal behavior, directly influencing heat generation under NIR irradiation [285, 286]. Beyond purely organic frameworks, hybridization with inorganic photothermal agents provides a robust route to improve efficiency and introduce complementary therapeutic or diagnostic functionalities. Embedding nanoparticles such as CuS, CuSe, FeS_2_, or polyoxometalates (POMs) into COF matrices yields composite nanosheets and core–shell structures that combine the porosity and surface tunability of COFs with the high photothermal performance of inorganic components. These hybrids exhibit strong photothermal effects both in vitro and in vivo and have been validated in tumor models for combined PTT/PDT or PTT‐enhanced chemo‐ and chemodynamic therapies (Figure 10A) [287, 293, 294].

The combination of PTT and PDT provides clear therapeutic advantages through reciprocal reinforcement mechanisms.

1. PTT‐induced hyperthermia transiently enhances blood flow, oxygen diffusion, and cell membrane permeability, thereby improving oxygen‐dependent Type II PDT in hypoxic tumor microenvironments.
2. Conversely, PDT‐mediated oxidative damage sensitizes tumor cells to thermal stress, lowering the temperature threshold required for ablation.

COFs are particularly effective for implementing photosensitizers and PTT centers within a single nanosystem, ensuring synchronized activation under a shared illumination wavelength. Representative systems have demonstrated significant in vivo tumor suppression through coordinated PDT/PTT mechanisms, confirming the design versatility of COF‐based carriers (Figure 10A) [275, 282].

Several complementary approaches have been used to optimize COF‐based PTT platforms:

1. Covalent incorporation or high loading of chromophores within COF backbones or pores minimizes aggregation‐caused quenching, thereby preserving both ROS generation (PDT) and light absorption (PTT) efficiency.
2. Hybrid inorganic–COF composites, where PTT nanoparticles (CuS, CuSe, FeS_2_) are anchored or encapsulated, combine efficient heat generation with COF‐mediated drug loading, targeting, or catalytic functions for chemodynamic therapy.
3. D–Architectures extend light absorption into the NIR region and enhance nonradiative relaxation, improving both PTT conversion and imaging potential [287, 295, 296].

Advanced COF‐based hybrids exhibit stimuli‐responsive or microenvironment‐adaptive behavior. POM–COF and metal‐decorated COFs can be engineered to respond to tumor‐specific cues, such as acidic pH or elevated hydrogen peroxide, thereby activating chemodynamic or PTT effects in tandem. These systems integrate multiple therapeutic pathways, PTT, PDT, and catalytic therapy, into single platforms capable of eliciting multimodal antitumor and immune responses [293, 294]. Multiple in vivo studies confirm that COF‐based and COF‐hybrid constructs achieve tumor regression and survival benefits in xenograft models when PTT is combined with PDT or chemotherapeutic co‐delivery [275, 282, 285].

From a translational standpoint, COF‐based PTT systems offer several distinct advantages:

1. Chemical modularity: facile tuning of optical absorption and heat conversion through monomer selection or post‐synthetic modification.
2. COF‐delivery capability: porous networks enable simultaneous incorporation of chemotherapeutics, immunomodulators, or photosensitizers for combination therapies.
3. Integrated imaging: intrinsic optical and photoacoustic contrast supports real‐time, image‐guided treatment.

Key challenges remain before clinical translation. These include improving tissue penetration depth (favoring NIR‐II absorbers), ensuring reproducible PTT conversion under physiological conditions, mitigating off‐target heating, and fully characterizing biodegradation and clearance of composite materials. Ongoing research focuses on extending absorption into the second NIR window, optimizing heat generation, biocompatibility trade‐offs, and integrating immune‐stimulatory payloads for synergistic PTT, immunotherapy [283, 286, 297].

### 7.2 Chemodynamic and Oxidation‐Based Therapies

Chemodynamic therapy (CDT) and related oxidation‐based modalities exploit in situ catalytic reactions to generate highly cytotoxic reactive species, particularly hydroxyl radicals (•OH), within the tumor microenvironment (TME) (Figure 10B). The canonical CDT mechanism involves Fenton or Fenton‐like reactions, in which transition metal ions such as Fe^2+^ or Cu^+^ catalyze the decomposition of endogenous hydrogen peroxide (H_2_O_2_) to produce •OH, thereby inducing oxidative damage to lipids, proteins, and nucleic acids. In the classical Fenton reaction, Fe^2+^ reacts with H_2_O_2_ to generate hydroxyl radicals (•OH) and Fe^3+^ (Fe^2+^ + H_2_O_2_ → Fe^3+^ + •OH + OH^−^), while Fenton‐like systems based on Cu^+^ or other transition metals follow analogous redox cycles [298]. COFs have recently emerged as versatile platforms for implementing and amplifying these redox chemistries. Their high porosity, tunable functionality, and modular architecture allow precise incorporation of metal centers, catalytic nanoparticles, or redox‐active enzymes, while maintaining spatial control over reaction microenvironments. Importantly, the porous architecture of COFs enables spatial confinement of catalytic centers, thereby enhancing local reactant concentration (e.g., H_2_O_2_) and facilitating electron transfer, thereby improving catalytic efficiency compared with homogeneous systems.

A growing number of experimental studies illustrate how COFs can be engineered to drive Fenton and Fenton‐like reactions within tumors. COF–metal composites or COF@metal sulfide hybrids have been shown to catalyze H_2_O_2_ decomposition, thereby elevating intratumoral •OH levels and producing significant tumor regression in preclinical models [288, 296, 299]. Anchoring Fe or Cu sites within COF matrices creates localized catalytic domains that enhance redox reactivity compared with free metal ions, while the porous scaffold improves tumor accumulation and retention. These systems combine high surface area, tunable metal coordination, and stability under physiological conditions, key attributes for achieving controlled radical generation in the TME.

An allied therapeutic mechanism, ferroptosis, exploits iron‐dependent lipid peroxidation to induce a regulated, non‐apoptotic form of cell death. Several COF‐based systems have been designed to couple metal‐catalyzed ROS generation with depletion of antioxidant defenses (e.g., glutathione), thereby promoting lipid peroxidation and ferroptotic signaling in cancer cells [288, 300]. This process is driven by iron‐dependent accumulation of lipid peroxides and depletion of GSH, leading to inactivation of glutathione peroxidase 4 (GPX4) and loss of cellular redox balance [301]. By combining metal delivery, ROS generation, and redox homeostasis disruption, COFs can effectively bias tumor cells toward ferroptosis pathways. This mechanism is attractive because ferroptosis can bypass specific resistance mechanisms of conventional chemotherapy and has been shown to elicit immunogenic cell death, potentially synergizing with immunotherapy.

Starvation therapy complements CDT by depriving tumor cells of essential nutrients while simultaneously generating oxidative stress. A common strategy involves loading glucose oxidase (GOx) or other oxidases into COF‐based nanocarriers. GOx catalyzes the oxidation of glucose to gluconic acid and H_2_O_2_, both starving tumor cells and supplying additional substrate for downstream Fenton reactions. COFs are well‐suited for enzyme encapsulation and cascade catalysis, as their porous frameworks protect enzymatic activity and permit co‐localization of GOx and metal catalysts. This spatial confinement enables sequential reactions (glucose oxidation → elevated H_2_O_2_ → Fenton reaction → •OH generation), which amplify oxidative stress in tumor tissues and improve therapeutic efficacy over single‐modality systems (Figure 10B) [289, 302, 303].

Recent studies have combined CDT, ferroptosis induction, and enzyme‐based starvation therapy into multifunctional COF architectures. For example, hybrid COFs that co‐deliver GOx and Fenton‐active metals, or that integrate or photocatalytic components, enable temperature‐enhanced Fenton kinetics and improved tumor perfusion, thus increasing the availability of O_2_ and H_2_O_2_. Such designs achieve synergistic tumor cell killing both in vitro and in vivo [290, 304, 305]. These combinatorial platforms address key limitations of classical CDT, such as limited endogenous H_2_O_2_, tumor hypoxia, or strong antioxidant defenses, while allowing spatiotemporal control over catalytic activation.

Most studies now include quantitative evaluation of ROS generation (using chemical probes and EPR spin trapping), lipid peroxidation and ferroptosis markers (e.g., malondialdehyde and GPX4 suppression), as well as in vivo biodistribution and efficacy analyses. Importantly, systemic toxicity and metal release profiles are routinely monitored to ensure safety [299, 300, 306].

Critical translational challenges remain, including:

1. Stability of catalytic centers and prevention of premature leaching.
2. Selective activation within the tumor microenvironment to avoid off‐target oxidative injury.
3. Control of metal dosage and biodistribution to minimize systemic toxicity.
4. Comprehensive mechanistic correlation between ROS generation, lipid oxidation, and therapeutic outcome.

Future progress will depend on establishing precise control over activation mechanisms, mitigating systemic metal toxicity, and developing quantitative assays linking ROS generation and lipid peroxidation to in vivo efficacy. As these gaps are addressed, COF‐based oxidation therapies are poised to evolve into a clinically relevant class of next‐generation catalytic nanomedicines.

### 7.3 COFs for Cancer Immunotherapy and Tumor Immune Modulation

Immunotherapy has transformed modern oncology by enabling the immune system to recognize and eliminate tumor cells. However, its clinical efficacy is still limited by low tumor immunogenicity, the immunosuppressive tumor microenvironment (TME), and systemic immune‐related adverse effects (Figure 10C). In this context, COFs have emerged as versatile platforms for cancer immunotherapy, serving as immunomodulatory carriers, adjuvant scaffolds, and multifunctional nanoplatforms that can be combined with chemotherapy, phototherapy, or CDT to enhance antitumour immune responses.

One of the most important mechanisms by which COF‐based systems promote antitumour immunity is the induction of immunogenic cell death (ICD). COF‐based PDT and PTT systems can trigger ICD, a form of regulated cell death associated with the release of damage‐associated molecular patterns (DAMPs), including calreticulin, ATP, and HMGB1. These signals promote dendritic cell recruitment and maturation, stimulate antigen presentation, and activate cytotoxic T lymphocytes, thereby converting immunologically “cold” tumors into “hot” tumors that are more responsive to immunotherapy.

Porphyrin‐ and phthalocyanine‐linked COFs have demonstrated potent PDT‐induced ICD in murine tumor models, with subsequent infiltration of CD8^+^ T cells and enhanced efficacy when combined with immune checkpoint blockade (e.g., anti‐PD‐1 therapy) [275, 282]. Similar effects have been observed for NIR‐absorbing donor–acceptor COFs that combine PDT and PTT functions. In these systems, ROS generation and local hyperthermia act synergistically to induce ICD and amplify systemic immune activation. Beyond ICD induction, the well‐defined porosity and modular surface chemistry of COFs make them attractive carriers for tumor‐associated antigens (TAAs) and immunostimulatory agents, such as CpG oligonucleotides and R848. Their ordered pore networks can protect sensitive biomolecules from premature degradation while enabling sustained release and co‐delivery with immune‐activating signals.

Recent examples demonstrate that amine‐ or imine‐linked nanoscale COFs can adsorb or covalently attach CpG motifs, leading to prolonged activation of toll‐like receptor 9 (TLR9) and enhanced antigen presentation by dendritic cells. The incorporation of redox‐ or pH‐sensitive linkages enables the release of adjuvants within endo‐lysosomal compartments, where immune signaling is initiated. In vivo, such formulations elicit robust humoral and cellular immune responses, validating COFs as synthetic adjuvant scaffolds that are comparable to, or surpass, traditional polymeric and liposomal systems (Figure 10C) [288]. The multifunctionality of COFs also enables integration of catalytic and immunomodulatory components within a single therapeutic system, allowing spatiotemporally coordinated multimodal therapy. For example, Fe‐ or Cu‐doped COFs that catalyze Fenton‐like reactions in CDT can not only induce oxidative tumor damage but also promote the release of tumor‐associated antigens and inflammatory cytokines, thereby priming antitumour immune responses [307].

Hybrid constructs combining PDT/PTT and checkpoint blockade therapies have demonstrated notable tumor suppression and prevention of metastasis in animal models. PTT heating enhances vascular permeability and antigen release, while concurrent checkpoint inhibition (using anti‐PD‐1 or anti‐CTLA‐4 antibodies) sustains immune activation, thereby establishing long‐term immune memory. A representative example is a porphyrinic COF integrated with polyoxometalates and loaded with DOX, which achieved triple‐modal chemo–photo–immunotherapy, inducing ICD, promoting dendritic cell maturation, and eliciting durable tumor rejection upon re‐challenge (Figure 10D) [290].

In addition to inducing ICD, COFs can be engineered to remodel the immunosuppressive TME and restore immune activity. Functionalization with catalytic moieties or enzymes can promote oxygen generation, as in catalase‐mimetic COFs, thereby alleviating hypoxia and improving ROS‐dependent immunogenicity during PDT. Other COF designs have been developed to deplete GSH or scavenge immunosuppressive molecules, thus altering the redox balance of the TME and improving immune recognition. Metal‐COF hybrids containing Mn, Fe, or Cu not only drive redox catalysis but also promote M1 macrophage polarization and dendritic cell activation, further amplifying antitumour immunity [308]. These immunometabolic effects emphasize that COFs can function not merely as passive carriers, but as active regulators of the tumor immune microenvironment. From a translational perspective, COF‐based immunotherapy is still at an early stage, but it shows considerable promise. Key advantages of COFs include:

1. High chemical tunability, which enables orthogonal functionalization with immunostimulants, therapeutic agents, and imaging probes.
2. Structural stability, combined with opportunities to introduce degradable or bioresponsive linkages.
3. Multimodal adaptability, which supports combination strategies such as PDT–PTT–CDT–immunotherapy.

Nevertheless, several challenges must still be addressed, including the need to ensure biodegradation and clearance, define immune safety and cytokine‐response profiles, and demonstrate reproducible efficacy in immunocompetent animal models. Comprehensive immunological evaluation, including cytokine profiling, T‐cell activation analysis, and assessment of immune memory, will be essential to understand how the physicochemical properties of COFs shape immune outcomes.

COFs have recently emerged as advanced platforms for cancer immunotherapy due to their highly tunable structure, large surface area, and capacity for multifunctional integration of therapeutic and immunomodulatory components [309]. A particularly important feature of COF‐based immunotherapeutic systems is that they can act as active “reactors” within the cancer–immunity cycle rather than as passive delivery vehicles. By integrating phototherapeutic, catalytic, and cargo‐delivery functions, COFs can simultaneously induce tumor cell death and coordinate immune activation through spatially and temporally controlled processes [309].

One key mechanism underlying COF‐mediated immunotherapy is the induction of immunogenic cell death (ICD). COF‐based systems, particularly those operating via PDT, PTT, or CDT, generate high levels of ROS, leading to oxidative stress, endoplasmic reticulum damage, and membrane disruption. These processes trigger the release of damage‐associated molecular patterns, including calreticulin exposure, ATP secretion, and HMGB1 release, which are essential for dendritic cell maturation and antigen presentation [310]. Experimental studies have demonstrated that structurally engineered COFs can enhance ICD efficiency by optimizing photophysical properties and ROS generation. For example, three‐dimensional COFs with tailored pore architectures and electronic structures exhibit significantly improved ROS production, thereby promoting stronger ICD responses and enhanced antitumor immunity [311].

Beyond ICD, COFs play a critical role in remodeling the immunosuppressive TME. Tumors are often characterized by hypoxia, elevated glutathione levels, and immunosuppressive cell populations that limit therapeutic efficacy. COF‐based nanoplatforms can alleviate hypoxia, deplete intracellular GSH, and promote oxidative stress, thereby shifting the TME toward a pro‐inflammatory and immunostimulatory state [309]. Such modulation enhances the recruitment and activation of immune effector cells, including cytotoxic T lymphocytes and natural killer cells, while simultaneously improving the efficacy of immune checkpoint blockade therapies. This ability to reprogram the TME positions COFs as powerful adjuvants in combination immunotherapy.

The intrinsic porosity and modularity of COFs make them highly effective carriers for immunomodulatory agents, including antigens, adjuvants, and immune checkpoint inhibitors. Their well‐defined pore structures enable high loading capacity, protection of sensitive biomolecules, and controlled release kinetics, which are essential for effective immune activation [253]. Importantly, COFs can facilitate antigen delivery to antigen‐presenting cells, particularly dendritic cells, enhancing antigen processing and cross‐presentation. This property enables the development of COF‐based nanovaccines that induce robust humoral and cellular immune responses. Additionally, nanoscale COFs can efficiently drain into lymph nodes, further enhancing immune priming.

Recent studies highlight the ability of COF‐based systems to induce alternative forms of immunogenic cell death, such as pyroptosis and ferroptosis, which further amplify immune responses. Pyroptosis, a highly inflammatory form of programmed cell death, is characterized by gasdermin‐mediated membrane rupture and cytokine release, leading to strong immune activation. COF‐based nanosystems have been specifically engineered to trigger pyroptosis and enhance antitumor immunity by converting immunologically “cold” tumors into “hot” tumors [312].

Similarly, ferroptosis, driven by iron‐dependent lipid peroxidation and redox homeostasis disruption, has been integrated into COF‐based therapeutic platforms. By incorporating catalytic metal centers or redox‐active components, COFs can promote lipid peroxidation and enhance immune‐mediated tumor suppression, offering synergistic effects with other therapeutic modalities [272, 313]. This capacity to induce multiple forms of immunogenic stress further distinguishes COFs from conventional nanocarriers and supports their use in combination immunotherapy. A major advantage of COFs is their ability to integrate multiple therapeutic modalities into a single platform. COF‐based systems combining PDT/PTT/CDT with immunotherapy have demonstrated synergistic effects, including enhanced tumor regression, inhibition of metastasis, and induction of systemic immune responses such as the abscopal effect [314].

These multifunctional platforms enable coordinated activation of innate and adaptive immunity, overcoming tumor heterogeneity and immune escape mechanisms. Furthermore, COFs can serve as delivery vehicles for immune checkpoint inhibitors or cytokines, enabling precise spatiotemporal control over immune activation. Despite this progress, the clinical translation of COF‐based immunotherapeutics will require better control over immune activation to avoid systemic inflammation, a deeper understanding of long‐term immunogenicity, and careful optimization of biodegradation and clearance. Future studies should therefore focus on establishing robust structure–immune function relationships, incorporating immunological design principles into COF synthesis, and developing standardized biological evaluation protocols. Such efforts will be critical for realizing the full potential of COFs as next‐generation platforms for cancer immunotherapy, nanovaccination, and combination immune modulation.

### 7.4 COF‐Based Antimicrobial and Wound‐Healing Platforms

COF's characteristics enable high loading of antimicrobial agents, controlled release, and surface modifications that resist bacterial adhesion and biofilm formation. Recent studies illustrate several complementary strategies for COF‐based antimicrobial design, including (i) incorporation of antibacterial ions or nanoparticles, (ii) controlled antibiotic or antiseptic release, (iii) embedding of photosensitizers for light‐activated disinfection, and (iv) integration of COFs into membranes or hydrogels that combine antimicrobial and hemostatic functions.

Embedding COFs into fibrous membranes or hydrogels provides dressings that release antimicrobial agents while maintaining a moist, pro‐healing microenvironment. A representative study demonstrated a curcumin‐loaded COF nanofibrous membrane with pH‐responsive release, strong antibacterial activity, and accelerated wound closure in vivo, highlighting how COF porosity supports sustained local delivery in wound settings [315]. Electrospun membranes incorporating cyclodextrin‐based COFs have achieved high antibiotic loading and release efficiency against *S. aureus* and *E. coli*, promoting faster tissue repair in full‐thickness wound models [316, 317]. These hybrid materials exemplify how the structural flexibility of COFs can be leveraged to improve topical drug delivery and wound healing outcomes.

Hybridization with inorganic agents provides synergistic antimicrobial effects through contact killing, catalytic ROS generation, and biofilm disruption. For instance, COF scaffolds decorated with Ag, Cu, or peroxidase‐like nanozymes exhibit potent antibacterial activity and enhanced antibiofilm effects compared with the inorganic agents alone, due to improved dispersion and sustained local release from the COF matrix (Figure 10E) [292, 318]. Several studies have exploited COF‐supported catalytic centers that convert endogenous or supplied substrates into bactericidal ROS, an especially promising strategy for chronic or infected wounds requiring sustained, localized disinfection [319, 320].

COF‐based photodynamic materials offer light‐triggered antibacterial activity and biofilm disruption through controlled ROS generation. Photosensitizer‐loaded COF membranes or nanosheets efficiently generate singlet oxygen under illumination, achieving selective eradication of both planktonic bacteria and mature biofilms while minimizing systemic exposure [321, 322]. Such photoactivated COF systems are particularly suitable for chronic or diabetic wounds, where illumination can be applied topically during dressing replacement, allowing on‐demand activation and localized action.

Beyond antimicrobial functionality, COFs have been incorporated into hemostatic and regenerative dressings that promote clot formation while delivering antibacterial or anti‐inflammatory agents. COF‐based composites accelerate hemostasis through rapid blood adsorption and clotting, coupled with sustained therapeutic release, leading to improved tissue regeneration in preclinical wound models [323, 324]. Similarly, COF‐functionalized sponges and cryogels act as physical hemostats with integrated antimicrobial function when doped with metal ions or antibiotics [319, 320]. Effective biofilm management is central to chronic wound therapy. Recent studies highlight that COF‐based coatings and membranes can significantly reduce biofilm biomass and viability, especially when combined with mild photodynamic activation or catalytic ROS generation [325, 326].

Across the literature, several translational themes emerge:

1. Controlled release: COFs mitigate burst release and maintain local drug levels above the minimum inhibitory concentration.
2. Stabilization: Labile antimicrobial agents (enzymes, photosensitizers) are protected from premature degradation within the COF network.
3. Surface control: Tunable chemistry discourages bacterial adhesion and supports tissue compatibility.

For clinical translation, the next essential steps include quantitative analysis of ion and drug leaching, demonstration of low cytotoxicity toward fibroblasts and keratinocytes, and validation in clinically relevant infection models (e.g., polymicrobial biofilms, diabetic wounds) [321, 327, 328].

### 7.5 Enzyme Immobilization and Biocatalytic Therapeutic Platforms Enabled by COFs

Enzyme immobilization within porous frameworks offers a powerful means to extend biocatalytic function into biomedical, pharmaceutical, and environmental domains. COFs’ attributes enable protection of enzymatic activity, efficient substrate diffusion, and precise control over the local microenvironment. Immobilization not only enhances enzyme resistance to proteolysis and thermal denaturation but also allows for enzyme recycling, spatial localization, and integration into therapeutic or detoxification systems [329]. In prodrug activation, the objective is to deliver enzymes directly to pathological sites, where they convert inactive precursors into pharmacologically active agents locally. Porous COF scaffolds are ideally suited for this purpose, as their high loading capacity and interconnected pore network enable efficient enzyme–substrate interaction while maintaining catalytic accessibility. Recent work has demonstrated that oxidoreductases and hydrolases immobilized within COF matrices retain their activity under physiological conditions, enabling on‐demand drug release at target sites (Figure 10F) [291].

Enzyme‐functionalized COFs have also been explored for detoxification therapies, where the goal is to neutralize toxic metabolites, ROS, or xenobiotics in situ. Peroxidases, laccases, and hydrolases immobilized within COF or COF‐like carriers show markedly improved half‐life and activity retention over multiple catalytic cycles. For example, catalase‐ and peroxidase‐loaded porous frameworks effectively decomposed hazardous chemicals and ROS while maintaining catalytic performance after repeated use [330, 331]. Such systems hold potential for therapeutic detoxification and oxidative stress management in biomedical contexts.

Enzyme immobilization in COFs can be achieved through physical adsorption, covalent attachment, entrapment during framework formation, or the formation of cross‐linked enzyme aggregates (CLEAs). Each approach presents trade‐offs in enzyme leakage, mass transfer, and activity retention. Studies using COF and COF‐analogous materials demonstrate that matching pore size and surface functionality to enzyme dimensions is crucial for balancing catalytic accessibility with structural stability [128, 332]. A distinguishing advantage of COFs over conventional carriers lies in their tunable internal microenvironment. Functionalized pores can stabilize specific enzyme conformations, adjust local pH and polarity, and facilitate electron transfer for redox‐active enzymes. These properties are especially relevant for oxidases and peroxidases involved in prodrug activation or detoxification cascades [333]. Computational modeling further supports the rational design of framework‐enzyme interfaces, predicting optimal diffusion dynamics and binding energies [334].

Key challenges for COF–enzyme systems include preventing enzyme leaching, minimizing substrate diffusion limitations, and ensuring the biocompatibility of degradation products. Recent progress in continuous‐flow reactors and COF–polymer hybrid composites offers promising solutions, enabling scalable fabrication and enhanced mechanical robustness while maintaining enzyme activity [335].

## 8 Diagnostics and Imaging

Across imaging modalities, COFs provide a platform for diagnostic and image‐guided medicine:

1. Fluorescent and persistent (afterglow) COFs enable bright, photostable probes with emerging persistence features.
2. Photoacoustic (PA) and PT COFs integrate energy conversion and therapy for real‐time monitoring.
3. Metal‐doped or composite COFs enhance imaging capabilities for MRI, CT, and PET, with potential for multimodal theranostics.

Fluorescence and afterglow emission represent two of the most direct and sensitive COF‐based imaging strategies. The primary challenge for luminescent COFs is suppressing nonradiative decay and aggregation‐caused quenching (ACQ), which commonly diminishes emission efficiency in highly conjugated solids. The seminal work by Li et al. demonstrated that careful structural design, particularly controlling interlayer stacking and framework rigidity, can yield solid‐state COFs with measurable photoluminescence [72]. Building on this, Yang et al. introduced non‐conjugated cyclohexane linkers into 2D imine COFs to disrupt π‐stacking, achieving photoluminescence quantum yields of up to 57% in the solid state, among the highest reported for imine COFs [336].

This design insight illustrates how structural modulation can preserve emissive states by reducing excitonic coupling between layers. For biological imaging, COFs embedding luminescent chromophores such as Ru(bpy) complexes have been developed, offering enhanced photostability and reduced dye aggregation in cellular environments [337]. Persistent luminescence, where emission continues after excitation ceases, is an emerging frontier. COF systems are being engineered to store excited charges or trap states that release photons gradually, thus improving signal‐to‐background ratios and imaging contrast. Although true long‐duration afterglow remains rare in organic frameworks, advances in trap‐state engineering, host–guest doping, and defect control are opening new directions [338]. Rigorous photophysical characterization, including absorption and emission spectra, quantum yield, lifetime (τ), and excitation dependence, is essential for validating emissive behavior. Recent mechanistic studies demonstrate ratiometric emission tuning through control of molecular structure and environment, providing design guidance for biological applications [339]. Key practical considerations include stability in aqueous and physiological environments, resistance to biomolecular quenching, and emission red‐shifting into the visible/NIR region, which enables deeper tissue penetration. Further progress will depend on balancing biostability, trap engineering, and chromophore integration to achieve bright, biocompatible, and persistent COF‐based probes.

**FIGURE 11:**
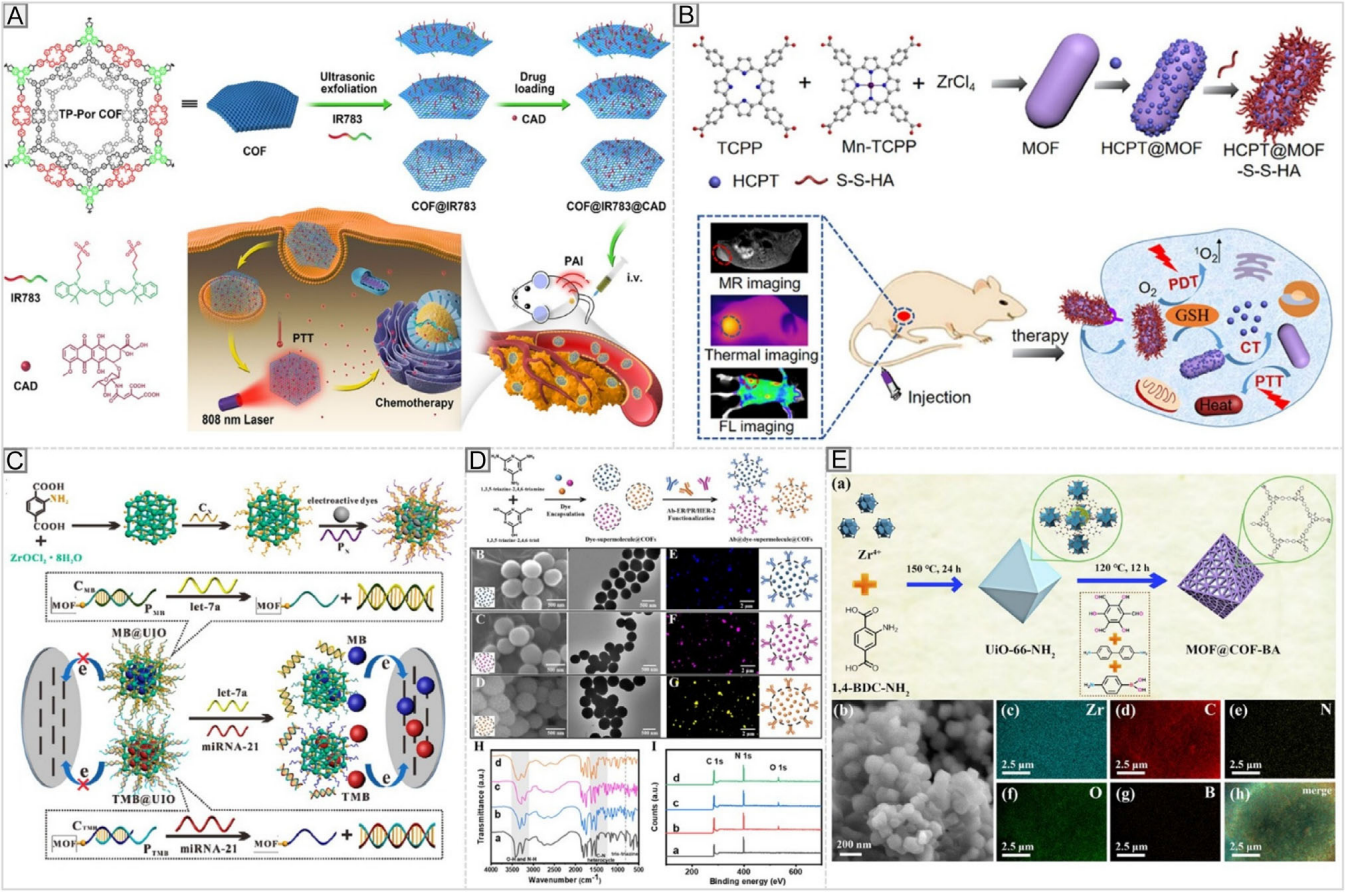
COF‐based platforms for biomedical imaging and sensing. (A) PA/PT imaging. Reproduced with permission from ref. [343]. Copyright 2019 American Chemical Society. (B) MRI/CT/PET multimodal contrast. Reproduced with permission from ref. [344]. Copyright 2022 American Chemical Society. (C) Electrochemical platforms. Reproduced with permission from ref. [345]. Copyright 2019 American Chemical Society. (D) Fluorescent platforms. Reproduced with permission from ref. [346]. Copyright 2024 American Chemical Society. (E) Colorimetric platforms. Reproduced with permission from ref. [347]. Copyright 2023 American Chemical Society.

PA and PT imaging convert absorbed light into acoustic or thermal signals, offering deep‐tissue penetration, high spatial resolution, and real‐time monitoring. COFs are inherently well‐suited to these modalities because their conjugated backbones and structural modularity support strong optical absorption and efficient nonradiative energy conversion.A landmark demonstration involved porphyrin‐based COF nanoparticles, which exhibited both photoacoustic contrast and photothermal conversion under NIR irradiation, achieving in vivo tumor imaging and ablation under a single light source [275]. Subsequent studies expanded this concept by hybridizing it with carbonaceous or polymeric segments to enhance NIR absorbance and thermal efficiency. For example, a COF composite with conjugated polymer domains provided high‐contrast imaging in food and biological matrices [340]. In biomedical contexts, COF–polymer and COF–inorganic composites have demonstrated stable photothermal conversion and strong PA signal generation under physiological conditions [130, 341, 342]. Hybrid architectures incorporating metal or CNs, such as COF–Au or COF–carbon composites, further amplify absorption and PA contrast (Figure 11A) [282, 343]. These systems support multifunctionality, combining imaging with therapy or delivery.

COFs can also serve as contrast platforms for MRI, CT, and PET through two main strategies:

i. Incorporation or chelation of imaging‐active metals (e.g., Mn^2+^, Gd^3+^, or radiometals) within the framework.
ii. Formation of composites embedding high‐Z nanoparticles or radiopaque species such as gold, bismuth, or iodinated clusters [348]. Metal‐functionalized COFs enhance relaxivity, enabling MRI visualization of biodistribution and tissue accumulation (Figure 11B) [344]. Radiolabel incorporation, using positron‐emitting isotopes coordinated to COF chelators, allows PET tracking of pharmacokinetics and target localization, supporting multimodal PET/optical/therapeutic workflows [349]. For CT contrast, high‐Z composites built from COF–Au, or COF–Bihybrids achieve strong X‐ray attenuation while retaining the COF's porosity for co‐loading of drugs or targeting ligands [350, 351]. A key advantage of COF contrast platforms is their native compatibility with multimodal and theranostic integration. Co‐loading magnetic centers, radiolabels, and chromophores enables MRI/PET/optical or CT/PA/PDT combinations for concurrent imaging and therapy [348, 352].

The development of COF‐based biosensors represents a rapidly advancing frontier in analytical biomedicine, driven by the exceptional porosity, chemical modularity, and electronic tunability of these materials. Recent progress has established COFs as versatile platforms across electrochemical, ratiometric fluorescence, and colorimetric sensing modalities, targeting biomarkers, metabolites, and therapeutic analytes under physiologically relevant conditions (Figure 11C).

Electrochemical COF biosensors exploit the high conductivity and functional tunability achievable through hybridization with metals or carbon nanostructures. For example, a conductive β‐ketoenamine COF deposited on carbon cloth enabled rapid, nanomolar‐level electrochemical detection of dopamine, owing to synergistic π–π interactions and well‐defined pore accessibility [353]. Similarly, COF@metal nanoparticle composites have demonstrated enhanced redox signal transduction, achieving ultrasensitive detection of cancer biomarkers such as carcinoembryonic antigen and prostate‐specific antigen [354]. The incorporation of redox‐active moieties, such as ferrocene, into COF backbones yields reproducible amperometric responses with excellent stability under physiological conditions [345].

Further enhancements arise from embedding bimetallic nanoparticles or conductive polymers within COF matrices, which enable multiplex analyte detection through differential pulse voltammetry and impedance spectroscopy [355, 356]. Flexible COF‐based electrodes have achieved real‐time, on‐skin detection of biochemical markers with high mechanical durability. Collectively, these results underscore the electrochemical versatility of COFs as redox‐active, stable, and customizable sensing platforms [357].

Fluorescent COF biosensors provide high photostability and tunable emission profiles through rational molecular design. Ratiometric sensing platforms, in particular, yield self‐calibrated signals that minimize artifacts from probe concentration or photobleaching. For example, a dual‐emissive COF combining porphyrin and anthracene units displayed excitation‐dependent ratiometric responses to ROS [358]. Likewise, boronate‐linked COFs coordinated with europium(III) complexes exhibited distinct dual‐emission bands for ratiometric detection of uric acid in serum [359].

Incorporating aggregation‐induced emission (AIE) chromophores into COFs further enhances signal stability and brightness. Nanoscale COFs containing AIE units have been utilized as intracellular pH probes, exhibiting high brightness and photostability [360, 361]. The ability to fine‐tune emission by linker choice or guest molecule incorporation, such as lanthanide coordination or dye encapsulation, has extended ratiometric fluorescence sensing to biologically relevant targets, including glucose, glutathione, and enzymatic activity [362]. These findings demonstrate how the molecular engineering of COFs enables stable and reproducible optical responses, even in complex biological media.

Colorimetric COF sensors rely on visible color changes resulting from catalytic or redox reactions, offering rapid and low‐cost detection without the need for specialized instrumentation. A representative example involves COF‐based nanozymes that catalyze the oxidation of chromogenic substrates, such as TMB, generating a color intensity proportional to the analyte concentration (Figure 11D) [346]. Incorporating hemin or Fe_3_O_4_ nanoparticles confers peroxidase‐like activity, enabling the sensitive detection of glucose and H_2_O_2_ [363, 364].

The integration of COFs into hydrogel or paper‐based sensing devices has advanced the development of portable, point‐of‐care diagnostics, enabling visual or smartphone‐based quantification (Figure 11E) [347, 365, 366]. Moreover, embedding noble‐metal nanoclusters within COFs amplifies plasmonic effects, enabling picomolar‐level colorimetric detection through intensified visible responses [363].

To provide a clearer overview of the current state of the field, representative examples of COFs applied in biomedical studies are summarized in Tables 1, 2 and 3. The tables highlight key aspects including framework composition, functionalization strategies, and corresponding therapeutic applications, illustrating the diversity of design approaches and the relationship between structural features and biological performance.

## 9 Disease‐Focused Case Studies (In Vivo Evidence Emphasized)

The clinical translation of COF‐based therapeutic platforms relies on disease‐centered studies that evaluate in vivo performance, mechanisms of action within the relevant microenvironment, and translationally meaningful endpoints such as tumor regression, survival, bacterial clearance, and hemostasis. This section summarizes representative in vivo evidence across oncology, infectious disease, and cardiovascular indications, illustrating how COF chemistry and architecture can be tailored to overcome disease‐specific biological barriers.

Cancer therapy remains the most extensively explored area for COF‐based therapeutics. Several in vivo studies have demonstrated that rationally designed COFs and COF hybrids can modulate the TME and achieve measurable antitumor effects. A recurring design strategy involves exploiting catalytic or stimuli‐responsive chemistries, such as CDT, ROS generation, and photothermal or photo‐oxidative modalities, to selectively amplify oxidative stress within tumors, leading to tumor cell death and immune activation. Multiple reports describe COF‐based systems that catalyze H_2_O_2_ decomposition via Fenton or Fenton‐like reactions, thereby increasing intratumoral ROS levels and suppressing tumor growth in vivo. Representative examples include metal‐functionalized or metal‐sulfide‐decorated COF composites, which generate cytotoxic hydroxyl radicals (•OH) and achieve significant tumor inhibition in murine models [367, 368, 369]. Other studies integrate cascade designs, co‐localizing oxidase enzymes or GOX analogs with metal catalysts, to elevate endogenous H_2_O_2_ levels and enhance CDT efficacy, producing superior tumor suppression compared with single‐modality controls [370, 371, 372, 373].

Beyond direct ROS generation, COF platforms are engineered to reprogram the TME, modify pH, deplete antioxidants such as glutathione, normalize hypoxia, and deliver immunomodulatory payloads to sensitize tumors to therapy. In vivo studies have shown that COF‐based systems can alleviate hypoxia (via PTT or catalytic oxygen generation), suppress reductive defenses, and promote ICD that augments antitumor immunity [374, 375]. More recent work integrates TME‐responsive drug release and multifunctional payloads to achieve dual local and systemic therapeutic effects, including reduced primary tumor burden and inhibition of metastasis [376, 377, 378].

COF‐based platforms have also demonstrated strong in vivo efficacy in models of bacterial and fungal infection, validating their potential as antimicrobial and antibiofilm agents. In bacterial infections, PDT, PTT, and nanozyme‐based COFs effectively eradicate bacterial loads in wound and implant infection models, leading to accelerated tissue repair and significantly reduced bacterial counts relative to controls [123, 379]. These systems typically employ light‐activated ROS generation or catalytic ROS production from COF‐anchored metal centers to disrupt biofilms and kill planktonic bacteria in situ.

For fungal infections, COF‐based delivery of antifungal drugs or COF‐mediated oxidative therapies has demonstrated a reduction in fungal burden in animal models [380]. Although in vivo antiviral applications remain limited, the chemical modularity and multifunctionality of COFs, including co‐delivery of antiviral drugs, immune agonists, or photochemical inactivation agents, present clear opportunities for future expansion into antiviral therapeutics. Across these infection models, in vivo endpoints such as microbial clearance, wound closure rates, and prevention of reinfection consistently demonstrate the translational promise of COF‐based anti‐infective strategies.

Emerging studies are expanding the application of COF‐based therapeutics to cardiovascular and thrombotic contexts, particularly where localized hemostasis, thrombus prevention, or targeted vascular drug delivery is required. In vivo data show that COF composites and surface coatings can influence thrombosis and vascular repair. For example, COF‐based injectable and surface‐applied composites incorporating anticoagulant or anti‐inflammatory agents have been shown to reduce thrombus formation and promote vascular remodeling in injury models [381]. Other studies describe COF‐containing hydrogels and coatings that achieve rapid hemostasis in hemorrhage models while providing antimicrobial protection, an advantageous combination for trauma and surgical applications [382, 383]. Furthermore, multifunctional COF‐hybrid nanoparticles designed for targeted delivery of thrombolytics or antiplatelet agents have shown favorable biodistribution and thrombus resolution in vivo [384].

## 10 Outlook, Future Directions, and Conclusions

Over the past decade, COFs have evolved from conceptual porous polymers into multifunctional materials with genuine potential for biomedical translation. The chemistry most relevant to biomedical use emphasizes modular covalent linkages, such as imine, β‐ketoenamine, boronate ester, hydrazone, and triazine, that provide tunable physicochemical properties, including hydrolytic stability, π–π conjugation, and reversible dynamic exchange for adaptive functionality. Rational selection of linkages and building units allows precise control over degradation rates, mechanical flexibility, surface charge, and hydrophilicity, all of which are key determinants of biocompatibility and safety. Future research should expand beyond classical condensation chemistries toward orthogonal, aqueous‐compatible, and bioorthogonal coupling reactions that yield frameworks stable under physiological conditions yet degradable through predictable enzymatic or hydrolytic pathways. A key research priority is the systematic evaluation of new linkage chemistries under physiological conditions, including their degradation products and potential immunogenicity, to identify candidates suitable for clinical development.

Understanding and controlling structure–property relationships remain central to predicting COF bio‐performance. Framework topology, pore size, and surface chemistry govern drug loading, diffusion, and interactions with biological interfaces. Electronic conjugation and donor‐acceptor design influence PTT and PDT activity, enabling control over energy transfer and ROS generation. Future efforts should focus on integrating computational modeling, in situ spectroscopy, and machine‐learning‐guided synthesis to build predictive correlations between atomic‐level structure and biological outcomes. These data‐driven approaches will guide the rational design of COFs tailored to specific therapeutic and diagnostic applications. Establishing standardized metrics for bioactivity and linking them to COF structural parameters will be essential to prioritize frameworks with the highest translational potential.

Synthesis and manufacturing under biomedical constraints represent the next major challenge for COF translation. Aligning COF production with GMP standards requires more than chemical optimization; it demands dedicated infrastructure, including cleanroom facilities, closed‐system reactors, validated solvent handling, and monitoring of in‐line particle size, porosity, and impurities. Integrating these processes with sterilization compatibility, protective formulation, and comprehensive analytical verification will be crucial for moving COFs from laboratory prototypes to clinically viable materials. Future development should prioritize scalable, reproducible, and environmentally benign synthetic routes, such as mechanochemical or solvent‐free polymerization, to minimize variability and meet regulatory expectations. Prioritizing translational research that couples synthetic optimization with regulatory‐compliant characterization will accelerate the pathway from bench to bedside.

The studies reviewed herein demonstrate that COFs offer an adaptable molecular platform for delivering small and fragile biomacromolecules. Their structural tunability, chemical stability, and functional surfaces enable precise loading, protection, and release control. Future work should correlate framework structure with release kinetics, integrate targeting ligands or stimuli‐responsive motifs, and perform systematic in vivo studies to evaluate biodistribution, degradation, and safety. COFs could progress from experimental carriers to clinically relevant drug delivery systems that combine stability, selectivity, and multifunctionality through these advances. Key priorities include designing COFs with predictable in vivo behavior and establishing robust animal models to assess therapeutic efficacy and toxicity prior to human studies.

COFs are poised to influence various biomedical fields, including precision drug delivery, gene editing, phototherapy, biosensing, and immunomodulation. However, significant translational challenges remain, including reproducible synthesis, long‐term biocompatibility, metabolic clearance, and regulatory approval. Addressing these issues through interdisciplinary collaboration among chemists, materials scientists, pharmacologists, and regulatory experts will be essential for advancing the field. Ultimately, COFs represent a class of porous materials and a versatile molecular design strategy that bridges chemical precision with biological complexity. Their continued evolution, driven by an understanding of structure–function, scalable synthesis, and translational rigor, will determine their role as next‐generation biomedical materials. Research priorities should include cross‐disciplinary consortia focused on bridging chemistry, biology, and clinical practice, ensuring that COF innovations meet both scientific and regulatory benchmarks.

The future of COFs lies in uniting molecular design with clinical translation. While the structural tunability of COFs is well established, transforming this versatility into safe, standardized, and reproducible materials remains a challenge. A major future direction involves developing quantitative structure–activity relationships that link framework topology, surface chemistry, and degradation behavior to cellular uptake, immune modulation, and clearance. Advanced tools such as molecular dynamics simulations, machine learning, cryo‐TEM, and synchrotron spectroscopy will accelerate the creation of predictive models that connect COF structure to biological performance. Prioritizing predictive frameworks that integrate computational and experimental insights will guide the rational design of COFs for specific clinical indications.

Another key direction will be chemistry‐driven innovation. Expanding beyond traditional imine and boronate linkages toward β‐ketoenamine, azine, amide, or triazine coupling can yield robust and biodegradable frameworks under physiological conditions. Incorporating dynamic covalent chemistry or supramolecular cross‐linking could enable reversible assembly, pH‐ or enzyme‐triggered disassembly, and on‐demand drug release. Furthermore, bioorthogonal synthetic strategies that operate in aqueous or physiological media will be crucial for directly interfacing COFs with biomolecules and living systems. Future studies should systematically compare these materials in terms of biocompatibility, degradability, and functional performance to define the most promising candidates for clinical translation.

GMP‐compatible, continuous, and sustainable manufacturing must become a priority at the translational scale. Implementing solvent‐free or mechanochemical synthesis with in‐line analytics for porosity, size, and purity will enhance reproducibility and environmental compliance. Establishing standardized protocols for sterilization, quality control, and biocompatibility testing will be crucial to advance COFs from proof‐of‐concept to preclinical and regulatory validation. Developing modular, scalable manufacturing platforms that can be rapidly adapted to different COF designs will accelerate translation and regulatory approval.

Future exploration of hybrid and hierarchical COF architectures offers another promising avenue. COF–polymer composites, COF–MOF heterostructures, and COF–biopolymer hybrids could combine structural precision with mechanical flexibility and biointegration. Incorporating responsive components such as peptides, aptamers, or redox‐active groups could enable precise control of therapeutic activation and biomolecular recognition. These hybrid systems may underpin next‐generation smart therapeutics with spatiotemporal control of drug release, photodynamic response, or immune modulation in complex biological environments. Establishing design principles and predictive rules for hybrid COF systems will be critical to translating their multifunctionality into safe and efficacious therapies.

Ultimately, the translational success of COFs will depend on comprehensive preclinical evaluation. Future studies should use standardized models to assess pharmacokinetics, biodegradation, immunogenicity, and long‐term safety. Integrating advanced imaging, multi‐omics, and quantitative toxicology will deepen understanding of how framework chemistry affects biodistribution and clearance. Broader in vivo testing across predictive disease models will be vital for validating efficacy and safety profiles. Defining a roadmap for preclinical assessment, including standardized assays and regulatory endpoints, should be considered a top priority for the field.

Ultimately, collaboration among chemistry, biology, and clinical science will be crucial to transforming COFs from sophisticated materials into practical medical solutions. Emerging areas, such as personalized nanomedicine, gene therapy, immunoengineering, and bioresponsive diagnostics, will benefit from COF platforms that combine structural precision with multifunctional adaptability. As our understanding of structure–function relationships deepens and synthetic control advances, COFs are expected to transition from laboratory innovations to essential components of the biomedical materials landscape, defining a new era of molecularly engineered therapeutics and diagnostics. Strategic prioritization of research areas, including predictive structure–activity relationships, hybrid material development, scalable manufacturing, and standardized preclinical testing, will be essential to realize the clinical potential of COFs. In particular, the field should now prioritize: (i) development of physiologically stable yet biodegradable linkage chemistries; (ii) establishment of quantitative structure–bio‐performance relationships; (iii) GMP‐compatible, scalable, and reproducible manufacturing routes; (iv) standardized analytical, toxicological, and immunological evaluation protocols; and (v) rigorous preclinical studies in predictive animal models that define pharmacokinetics, biodegradation, clearance, and long‐term safety. Together, these priorities provide a practical roadmap for advancing COFs from promising laboratory materials to clinically relevant biomedical platforms.

## Conflicts of Interest

The authors declare no conflicts of interest.

## Data Availability

The data that support the findings of this study are available from the corresponding author upon reasonable request.
